# Hybrid biochar/layered double oxide for phosphate and humic acid removal from water: dominant role of memory-effect-driven anion intercalation

**DOI:** 10.1039/d6ra03448a

**Published:** 2026-08-20

**Authors:** Racha Kara, Abdelkader Ouakouak, Lynda Hecini, Dhirar Ben Salem, Noureddine Rouahna, Fouzia Touahra, Nesrine Zenidi, David Houivet, Nguyen Trong Anh, Hai Nguyen Tran

**Affiliations:** a Research Laboratory in Subterranean and Surface Hydraulics, University of Biskra PO Box 145 Biskra 07000 Algeria racha.kara@univ-biskra.dz; b Faculty of Technology, University of El Oued PO Box 789 El Oued 39000 Algeria; c Scientific and Technical Research Center for Arid Areas (CRSTRA) M.B. 1682 Biskra 07000 Algeria; d Research Centre in Analytical Chemistry and Physics (CRAPC) BP 248 Algiers 16004 Algeria; e Laboratory of LARGHYDE, University of Biskra PO Box 145 Biskra 07000 Algeria; f Laboratoire Universitaire des Sciences Appliquées de Cherbourg (LUSAC), ER 4253, Université de Caen-Normandie BP 78 50130 Cherbourg-en-Cotentin France; g Faculty of Science and Food Technology, Lac Hong University Vietnam; h Center for Energy and Environmental Materials, Institute of Fundamental and Applied Sciences, Duy Tan University Ho Chi Minh City 70000 Vietnam trannguyenhai@duytan.edu.vn; i Faculty of Environmental and Chemical Engineering, Duy Tan University Da Nang City 50000 Vietnam

## Abstract

A biochar/layered double oxide (LDO) composite was successfully developed for the simultaneous removal of phosphate (P) and humic acid (HA) from aqueous solutions. The composite was synthesised by *in situ* co-precipitation of Mg^2+^ and Al^3+^ salts to form a Mg/Al-layered double hydroxide (Mg/Al-LDH) on palm fibre-derived biochar, followed by calcination at 500 °C. X-ray diffraction confirmed the successful formation of crystalline Mg/Al-LDH with a basal spacing (*d*_003_) of 0.755 nm. The specific surface areas of PF-biochar, biochar/LDH, and biochar/LDO were 465.2, 268.3, and 241.2 m^2^ g^−1^, respectively. The Langmuir maximum adsorption capacities for P and HA followed the order: biochar/LDO (28.6 and 152.9 mg g^−1^) > biochar/LDH (15.0 and 112.7 mg g^−1^) > PF-biochar (7.40 and 74.1 mg g^−1^). Thermodynamic analysis showed that the adsorption of P (Δ*H*° = 15.92 kJ mol^−1^) and HA (Δ*H*° = 13.56 kJ mol^−1^) was endothermic and predominantly governed by physical interactions. XRD analysis of biochar/LDO revealed that the basal spacing of the reconstructed LDH increased from 0.741 nm after rehydration in water to 0.882 nm and 1.147 nm following phosphate and HA adsorption, respectively, demonstrating anion intercalation associated with the memory effect. In addition to the memory effect, phosphate adsorption involved electrostatic attraction and surface complexation, whereas humic acid adsorption additionally involved electrostatic attraction, hydrogen bonding, and π–π interactions. The estimated direct operating cost for preparing the biochar/LDO composite was approximately 1.86 (US$ per kg). These findings demonstrate that the biochar/LDO composite is a promising low-cost and reusable adsorbent for the simultaneous removal of organic and inorganic contaminants from wastewater.

## Abstract 2

Biochar/layered double oxide (LDO) enables phosphate and humic acid adsorption through memory-effect-driven structural reconstruction and anion intercalation.

## Graphical abstract

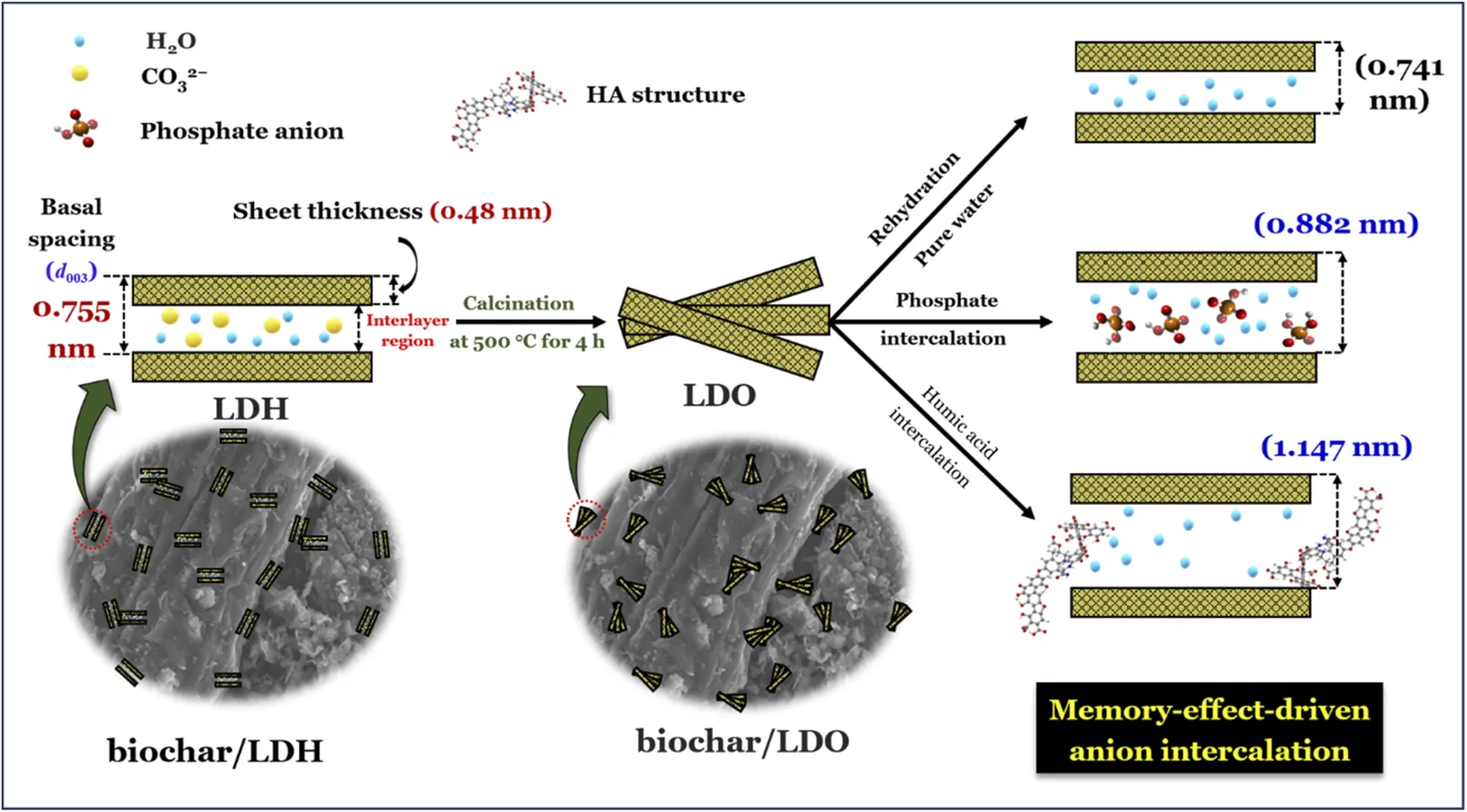

## 1. Introduction

Wastewater treatment has become an increasingly important environmental challenge due to the continuous discharge of organic and inorganic contaminants into aquatic environments, posing serious risks to human health and ecosystem sustainability.^1^ Phosphorus is an indispensable nutrient required for numerous metabolic processes, growth, and development in all living organisms.^2,3^ However, excessive phosphorus released from domestic, agricultural, and industrial wastewater is a primary cause of eutrophication in aquatic ecosystems.^4,5^ Eutrophication may occur when phosphorus concentrations exceed approximately 0.1 mg L^−1^, promoting harmful algal blooms and reducing dissolved oxygen levels, ultimately degrading water quality.^6–8^ Humic acid (HA), one of the major components of natural organic matter (NOM), is a heterogeneous, high-molecular-weight, and poorly biodegradable organic macromolecule generated during the decomposition of plant and animal residues.^9,10^ It is formed through the chemical and biological decomposition of organic materials, such as plant and animal residues.^1^ The presence of HA in water causes undesirable colour, odour, and taste and influences the migration of heavy metals.^11^ Although HA is not considered highly toxic, it readily participates in reactions that generate secondary contaminants. More importantly, chlorination of HA during drinking water treatment can form carcinogenic disinfection by-products, including trihalomethanes.^12,13^

Phosphate (P) and HA commonly coexist in municipal wastewater, agricultural runoff, and natural surface waters^9,14^ Their coexistence not only deteriorates water quality but also complicates treatment processes because humic substances may compete with phosphate for active adsorption sites, thereby influencing adsorption behaviour and removal efficiency. Consequently, the simultaneous removal of phosphate and HA has attracted increasing attention because binary-solute systems more closely represent practical water treatment conditions than conventional single-solute studies.^9,15^

Phosphate is a major inorganic anionic pollutant responsible for eutrophication,^4,8^ whereas HA is the predominant organic anionic component of natural organic matter that deteriorates water quality and serves as a precursor for carcinogenic disinfection by-products during chlorination.^13,16^ Investigating the simultaneous adsorption of P and HA also enables the evaluation of adsorption interactions under binary-solute conditions, providing a more realistic assessment of adsorbent performance.^14^ Therefore, the simultaneous removal of these representative inorganic and organic pollutants is of considerable practical and environmental importance. Several treatment technologies, including precipitation, ion exchange, membrane filtration, biological treatment, and adsorption, have been developed for their removal.^2^ Among these technologies, adsorption has been widely recognised as one of the most promising approaches due to its operational simplicity, high removal efficiency, low operating costs, and potential for adsorbent regeneration and reuse.^9,17,18^

Biochar is a porous carbonaceous material produced by the pyrolysis of biomass under oxygen-limited conditions and has attracted considerable attention as a sustainable adsorbent for water treatment.^13,15,19–21^ Owing to its abundant surface functional groups, high porosity, and excellent chemical stability, biochar has emerged as a promising adsorbent for removing a wide range of water contaminants.^17,22^ These properties make biochar particularly effective for HA removal, as HA contains abundant oxygen-containing functional groups, including –C=O, –OH, and –COOH.^13^ Nevertheless, the negatively charged surface and limited anion-exchange capacity of biochar considerably restrict its affinity for anionic pollutants, such as phosphate, owing to electrostatic repulsion.^4^ Consequently, pristine biochar generally exhibits a relatively low phosphate adsorption capacity, typically below 5 mg g^−1^.^23^ For instance, biochars derived from wood chips, corn cobs, and garden wood waste exhibited phosphate adsorption capacities of only 0.296, 0.036, and 0.132 mg g^−1^, respectively.^22^

Among various modification strategies, layered double hydroxides (LDH) have emerged as among the most effective materials due to their high anion-exchange capacity, tunable chemical composition, large specific surface area, and unique layered structure, which can accommodate a wide variety of anionic species.^3,4,8,17,24^ The general chemical formula of LDH is [M_1−*x*_^2+^ M_*x*_^3+^(OH)_2_]^*x*+^ [A_*x*/*n*_·H_2_O]^*n*−^; where M^2+^ and M^3+^ represent divalent (*e.g.*, Zn^2+^, Mg^2+^, Mn^2+^, Fe^2+^, Ni^2+^, and Ca^2+^) and trivalent (*e.g.*, Fe^3+^, Al^3+^, Co^3+^ and Cr^3+^) metals, respectively; *A*^−*n*^ represents the anion within the interlayer space, with a valence of *n* (*e.g.*, CO_3_^2−^, SO_4_^2−^, Cl^−^, and OH^−^); and *x* is the molar ratio of [M^2+^]/([M^2+^] + [M^3+^]).^16,17,25,26^ Because of this unique structure, LDHs exhibit excellent adsorption performance toward negatively charged contaminants through surface adsorption and anion exchange. Moreover, calcination of LDH generally results in higher adsorption capacities than those of their pristine counterparts.^3,4,24^ For example, Rouahna^3^ reported that the Langmuir maximum adsorption capacity of Zn/Al-LDO for orthophosphate (132.6 mg g^−1^) was substantially higher than that of Zn/Al-LDH (78.9 mg g^−1^), highlighting the beneficial effect of calcination on adsorption performance.

Calcination transforms LDH into layered double oxides (LDO), producing metastable mixed oxides that can reconstruct their original layered structure when exposed to water containing suitable anionic species. This unique reconstruction phenomenon, commonly known as the memory effect, is one of the most important characteristics distinguishing LDOs from conventional metal oxides.^4,24,27,28^ During rehydration, anionic species are incorporated into the regenerated LDH interlayers to restore charge neutrality, a process known as anion intercalation. Previous mechanistic studies have demonstrated that structural reconstruction proceeds *via* dissolution–rehydration–recrystallisation of the calcined oxide, regenerating the LDH layered structure.^10,29^ This reconstruction mechanism provides the fundamental basis for LDOs' superior adsorption performance toward anionic contaminants.^4,5,16,18^

Therefore, combining LDO with biochar offers a synergistic strategy that integrates LDO's high adsorption capacity with biochar's porous carbon framework.^5,8,30^ For example, Yang *et al.*^19^ prepared biochar composites containing Ni/Fe-LDH, Mg/Al-LDH, and Zn/Al-LDH, all of which exhibited enhanced phosphate adsorption compared with pristine biochar. Among these LDHs, Mg/Al-LDH has received particular attention because of its excellent hydrophilicity, structural stability, and pronounced memory effect after calcination, making it an attractive precursor for preparing highly efficient LDO-based adsorbents.^5,8,25^ These composites, therefore, show considerable potential for the simultaneous removal of inorganic and organic anionic contaminants (*i.e.*, phosphate and HA) from wastewater.

Despite these advances, previous studies have primarily focused on the adsorption of individual inorganic or organic pollutants.^2,5,16,19,25,26^ The structural evolution of biochar-supported LDO samples during the simultaneous removal of coexisting phosphate and humic acid has received considerably less attention. More importantly, the contribution of memory-effect-driven anion intercalation to binary adsorption systems has not been systematically clarified, and the adsorption interactions between phosphate and humic acid remain poorly understood. Addressing these issues is essential for elucidating the adsorption mechanism of LDO-based composites and for the rational design of efficient adsorbents for complex wastewater matrices.

In this study, a biochar/LDO composite was synthesised by calcining a biochar/LDH precursor and evaluated for the simultaneous removal of phosphate and humic acid from aqueous solutions. Particular emphasis was placed on correlating the structural transformation from LDH to LDO with adsorption performance. The adsorption mechanism was systematically investigated using XRD and FTIR analyses to elucidate the role of memory-effect-driven anion intercalation. Binary adsorption experiments were further performed to clarify the interactions between phosphate and humic acid under conditions representative of practical water treatment systems. Finally, the composite's preparation cost was estimated to evaluate its economic feasibility and practical application.

## 2. Materials and methods

### 2.1. Reagents and materials

Palm fibres (PF) were collected from the campus of Mohamed Khider University, Biskra, Algeria, and used as the biomass precursor for biochar preparation. The chemicals used in this study and their analytical grades are listed in Section S1 of the SI.

### 2.2. Preparation of biochar

The PF stalks were cut into small pieces (<2 cm), thoroughly rinsed with distilled water, and oven-dried at 110 °C for 48 h. The dried material was ground, sieved (0.1–1.0 mm), and pyrolysed in a muffle furnace (DAIHAN Scientific FHX-05) at 600 °C for 2 h under oxygen-limited conditions. The resulting biochar was soaked in 0.1 M HCl, washed with distilled water until the filtrate reached pH 6–7, and then dried at 110 °C for 24 h. Finally, the biochar was sieved to a particle size of 0.08–0.20 mm and designated PF-biochar.

### 2.3. Preparation of biochar/LDH composite

The biochar/LDH composite was synthesised by *in situ* co-precipitation of Mg^2+^ and Al^3+^ ions (Mg^2+^/Al^3+^ molar ratio = 2 : 1) on the surface of PF-biochar. This molar ratio was selected based on the previous suggestions.^5^ Two precursor solutions were prepared. The first (metal solution) was obtained by dissolving 6.71 g of magnesium chloride and 3.98 g of aluminium chloride in 50 mL of distilled water. The second (alkaline solution) consisted of 50 mL containing 2 M NaOH and 1 M Na_2_CO_3_.

Both solutions were stirred separately at room temperature for 30 min until completely dissolved. They were then simultaneously added dropwise to 20 mL of distilled water containing 5 g of PF-biochar. The suspension pH was maintained at 9.5–10.5 throughout the coprecipitation process by the simultaneous addition of the alkaline solution. The suspension was subsequently aged at 60 °C under continuous stirring (300 rpm) for 18 h to promote the crystallisation of Mg/Al-LDH. The resulting precipitate was collected by filtration, washed repeatedly with distilled water, and dried at 80 °C for 24 h. The resulting composite was designated biochar/LDH (Fig. S1c).

### 2.4. Preparation of biochar/LDO composite

The biochar/LDH composite was calcined at 500 °C for 4 h to produce approximately 5.1 g of the biochar/LDO composite. The obtained material was sieved to a particle size of 0.08–0.20 mm, designated biochar/LDO, and stored in airtight containers for subsequent adsorption experiments (Fig. S1c).

For comparison, two reference materials, Mg/Al-LDH and Mg/Al-LDO, were synthesised in the absence of PF-biochar, as illustrated in Fig. S1c. Mg/Al-LDH was prepared using the same co-precipitation method described above, whereas Mg/Al-LDO was obtained by calcining Mg/Al-LDH at 500 °C.

### 2.5. Materials' characterization

Details of the analytical instruments, operating conditions, and the pH drift method used for material characterization are provided in Section S2 of the SI.

### 2.6. Batch adsorption experiments

Batch adsorption experiments were conducted to evaluate the removal of phosphate (P) and humic acid (HA) from aqueous solutions using the biochar/LDO composite. Stock solutions of P (1000 mg L^−1^) and HA (500 mg L^−1^) were prepared in distilled water and diluted to the required concentrations. Unless otherwise stated, each experiment was performed in a 50 mL glass flask containing 50 mL of solution and 50 mg of adsorbent. The suspensions were shaken at 200 rpm for the required contact time while maintaining the desired temperature. After adsorption, the mixtures were filtered through a 0.45 µm membrane filter to separate the adsorbent particles from the aqueous phase, thereby terminating adsorption and yielding a clear solution for accurate UV-vis analysis, free of suspended particles. The residual phosphate concentration was determined using the heptamolybdate blue method with a UV-vis spectrophotometer (Optizen 2120 UV) at 880 nm, whereas the HA concentration was measured using a photoLab 7600 UV-vis spectrophotometer at 254 nm.

The experimental conditions, including solution pH, initial concentration, contact time, and temperature, are summarised in Table S1. Batch adsorption isotherms were also determined for PF-biochar and biochar/LDH to compare their adsorption performances with that of the biochar/LDO composite.

To investigate the competitive adsorption of P and HA, binary adsorption experiments were performed. Equal volumes (25 mL) of P and HA solutions containing 20 or 80 mg L^−1^ were mixed to obtain final solutions containing 10 or 40 mg L^−1^ of each solute in a total volume of 50 mL. After adsorption, the suspension was immediately filtered through a 0.45 µm membrane to completely separate the adsorbent from the aqueous phase, terminate further adsorption, and obtain a clear filtrate for subsequent UV-vis analysis. The filtrate was then divided into two aliquots. One aliquot was analysed directly for HA by measuring the absorbance at 254 nm, whereas the second aliquot was reacted with ammonium molybdate reagent prior to phosphate determination at 880 nm using the molybdenum blue method. The two analytes were therefore determined independently using different analytical principles and detection wavelengths, minimising potential analytical interference. Because the biochar was produced at 600 °C and subsequently calcined at 500 °C during composite preparation, the release of dissolved organic matter from the composite was expected to be minimal, consistent with previous studies on highly carbonised biochars.^20,21^

### 2.7. Reusability of biochar/LDO composite adsorbent

The reusability of the biochar/LDO composite was evaluated through successive adsorption–desorption cycles. For each adsorption experiment, 0.5 g of adsorbent was added to 500 mL of solution containing 30 mg L^−1^ of P or 100 mg L^−1^ of HA at the equilibrium pH (pH_eq_) values of 5 and 7, respectively. The suspensions were stirred for 4 h. After each adsorption cycle, the adsorbent was separated by filtration, dried, and directly reused in the subsequent cycle at a constant adsorbent dosage of 1 g L^−1^. The adsorption cycles were continued until the removal efficiency decreased below 50%. The exhausted adsorbent obtained from the final adsorption cycle was then immersed in 0.1 M NaOH under continuous stirring for 4 h to regenerate the material. After regeneration, the adsorbent was thoroughly rinsed with distilled water, dried, and subjected to a subsequent adsorption experiment to evaluate its regenerated adsorption performance.

The adsorption efficiencies of P and HA during the regeneration experiment were determined using eqn (1).1
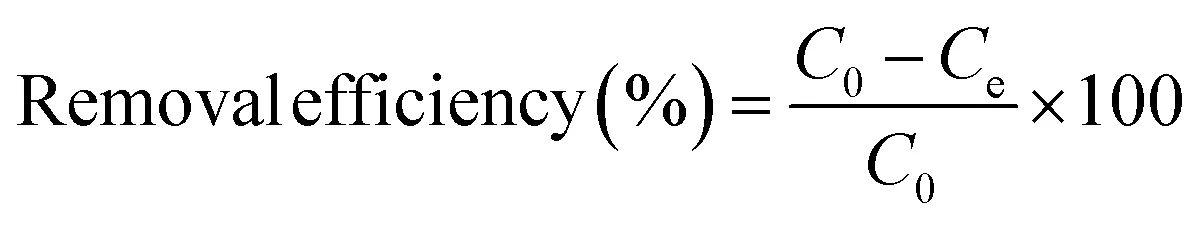
where *C*_0_ and *C*_e_ (mg L^−1^) denote the initial and equilibrium concentrations of P or HA during the desorption tests, respectively.

### 2.8. Analysis of adsorption data

The detailed analysis of adsorption data is presented in Section S3 (SI).

## 3. Results and discussion

### 3.1. Characterization of the prepared materials

#### 3.1.1. Crystal structure

**Fig. 1:**
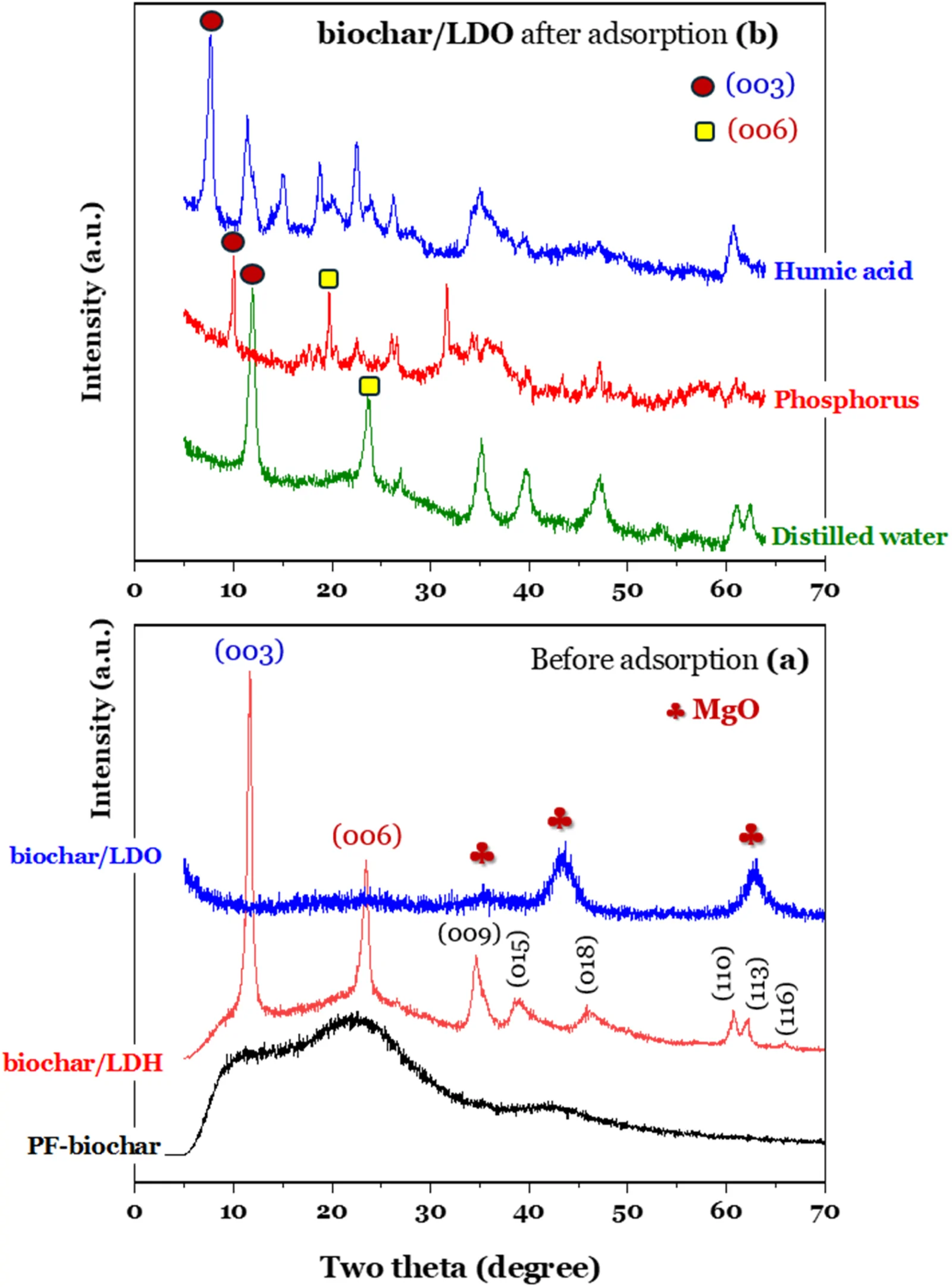
XRD patterns of (a) PF-biochar, biochar/LDH, and biochar/LDO, and (b) biochar/LDO after rehydration in distilled water and adsorption of phosphate and humic acid.

The XRD patterns of the prepared materials are presented in Fig. 1 and S2. As shown in Fig. 1a, PF-biochar exhibited a predominantly amorphous structure, as evidenced by the absence of distinct crystalline diffraction peaks.^2,4,13,17,25^ In contrast, Mg/Al-LDH displayed the characteristic diffraction pattern of a well-crystallised hydrotalcite-like structure (Fig. S2a).^4,27,31^ The characteristic diffraction peaks were consistent with the standard hydrotalcite pattern (PDF No. 01-089-0461; Section S4)^11,24^ observed at 2*θ* = 11.3°, 22.8°, 34.4°, 38.4°, 45.3°, 60.4°, 61.7°, and 65.5° were assigned to the (003), (006), (101), (015), (018), (110), (113), and (116) crystal planes, respectively (Fig. S2a).^4,28,31,32^ Among them, the intense reflections at 11.3°, 22.8°, and 34.4° were characteristic of Mg/Al-LDH,^33^ whereas the weaker peaks at 38.4°, 45.3°, 60.4°, 61.7°, and 65.5° were attributed to γ-AlOOH (boehmite).^26^

Likewise, biochar/LDH (Fig. S2b) exhibited diffraction peaks similar to those of pure Mg/Al-LDH (Fig. S2a), confirming the successful formation of the Mg/Al-LDH phase on the biochar surface through the *in situ* coprecipitation of Mg^2+^ and Al^3+^ salts. Although PF-biochar is predominantly amorphous (Fig. 1a), the crystalline Mg/Al-LDH phase (Fig. S2a) was successfully formed on its surface (Fig. 1a or S2b). Consequently, the diffraction pattern of the biochar/LDH composite exhibited the characteristic reflections of Mg/Al-LDH superimposed on the amorphous background of biochar. A similar finding was found for similar compositions: biochar/MgAl-LDH,^11,13,25^ activated carbon/MgFe-LDH,^2^ and biochar/CaFe-LDH composites.^17^

The structural parameters of biochar/LDH were further evaluated using the lattice constants *a* (related to the Mg^2+^/Al^3+^ cation ratio) and *c* (associated with the stacking sequence of the hydroxide layers). These parameters were calculated from the (110) and (003) reflections using the relationships *a* = 2*d*_110_ and *c* = 3*d*_003_.^5,29^ The calculated values of *a* = 0.304 nm and *c* = 2.27 nm were comparable with those previously reported for Mg/Al-LDH.^8,10^ The basal spacing (*d*_003_) was calculated to be 0.755 nm from the (003) reflection at 2*θ* = 11.7° (Fig. S2b) using Bragg's law, which is slightly smaller than that of pure Mg/Al-LDH (0.782 nm; Fig. S2a) and is consistent with previously reported values.^4,10,24,27^ The obtained *d*_003_ value is characteristic of carbonate-intercalated Mg/Al-LDH and confirms the successful formation of a well-ordered layered structure.^13^

After calcination, the XRD patterns of Mg/Al-LDO (Fig. S2c) and biochar/LDO (Fig. S2d) showed the disappearance of the characteristic Mg/Al-LDH reflections at approximately 11°, 23°, and 34°, indicating the collapse of the layered hydrotalcite structure and its transformation into LDO.^27,28^ Diffraction peaks at approximately 35°, 43°, and 62°, corresponding to the (111), (200), and (220) planes of MgO (JCPDS card no. 00-004-0829; Section S5),^24^ became clearly visible, confirming the formation of oxide phases (as the dominant crystalline phase) during calcination.^5,8,31,32^ Notably, the Al_2_O_3_ phase was not detected in the XRD spectrum of Mg/Al-LDO (Fig. S2c), suggesting that Al^3+^ ions remained highly dispersed within the periclase-like MgO structure rather than forming a separate crystalline alumina phase.^31^

The disappearance of the characteristic LDH reflections together with the appearance of MgO reflections confirms the complete transformation of Mg/Al-LDH (Fig. S2a) into Mg/Al-LDO (Fig. S2c) during calcination. This transformation resulted from the dehydroxylation and decarbonation of the LDH layers.^4,27^ Consequently, a metastable oxide structure was formed. Upon rehydration in the presence of water and anionic species, this metastable structure reconstructed the original LDH layers, giving rise to the characteristic memory effect.^10,16,29^ Furthermore, the broad diffraction band centred at approximately 20–25° remained visible after calcination (Fig. S2d), indicating that the amorphous carbon framework of biochar was largely preserved during LDO formation. This preserved carbon matrix provides structural support for the oxide phase while maintaining the composite architecture during the LDH–LDO transformation.

To further verify the structural transformation induced by calcination and the successful incorporation of the oxide phase into the biochar matrix, the morphology of the prepared materials was subsequently investigated by SEM.

#### 3.1.2. Surface morphology and elemental composition

**Fig. 2:**
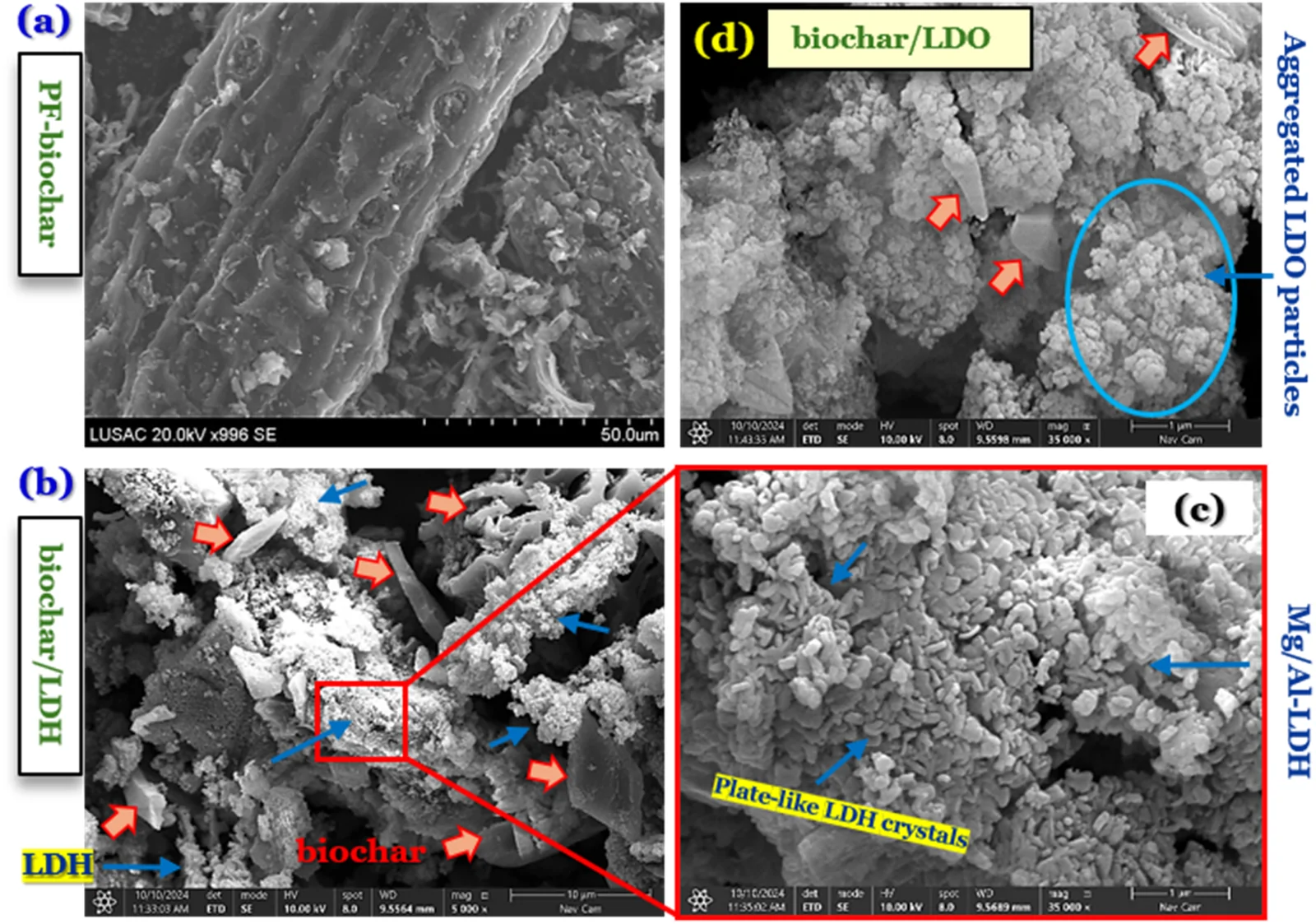
SEM micrographs of (a) PF-biochar, (b) and (c) biochar/LDH showing the Mg/Al-LDH nanosheets deposited on the biochar surface, and (d) biochar/LDO.

The surface morphology of PF-biochar, biochar/LDH, and biochar/LDO was characterized using SEM-EDS, and the corresponding micrographs are shown in Fig. 2. PF-biochar exhibited a relatively rough surface with longitudinal cracks (Fig. 2a). In contrast, the SEM image of pure Mg/Al-LDH (Fig. S3a) displayed the typical lamellar morphology of hydrotalcite-like materials.^24^ The particles consisted of stacked plate-like crystallites aggregated into irregular flower-like clusters,^4,27^ which are characteristic of well-crystallised hydrotalcite-like LDH^31,33^ and consistent with the XRD results (Fig. S2a). This morphology is typical of LDHs synthesised by coprecipitation, in which nanosheets aggregate to minimise surface energy.^10^

After calcination, Mg/Al-LDO (Fig. S3b) retained the overall aggregated morphology, whereas the original layered structure became considerably less distinct. The particles appeared more compact and densely packed, reflecting the collapse of the hydroxide layers caused by dehydroxylation and decarbonation during calcination.^5^ The disappearance of the well-defined lamellar structure further supports the structural transformation of LDH into LDO observed by XRD.^4,10,16,29^ This morphological evolution is consistent with the transformation of Mg/Al-LDH (Fig. S2a) into oxide phases identified by XRD analysis (Fig. S2c).

After Mg/Al-LDH deposition, the biochar/LDH composite developed a typical layered morphology, composed of stacked flakes and irregular aggregates (Fig. 2b). These aggregates indicate the presence of overlapping LDH sheets (Fig. 2c). A similar heterogeneous structure morphology of LDH-based composites was observed elsewhere.^11,30^ These observations confirm that Mg/Al-LDH was deposited and uniformly anchored on the PF-biochar surface through the *in situ* co-precipitation process. The intimate contact between the LDH particles and the biochar surface suggests strong interfacial integration, which is expected to facilitate subsequent structural reconstruction during the LDH–LDO transformation.^10^ Furthermore, the EDS spectrum of biochar/LDH (Fig. S4) showed dominant signals for C (59.8 at%) and O (34.1 at%), together with Mg (3.4 at%) and Al (1.4 at%). The high C and O contents can be attributed to the carbon matrix of PF-biochar, oxygen-containing surface functional groups, interlayer carbonate (CO_3_^2−^) anions, and hydroxyl groups associated with the LDH structure.

After calcination, the layered Mg/Al-LDH structure transformed into Mg/Al-LDO, yielding more compact and aggregated particles on the biochar surface (Fig. 2d). The morphology of biochar/LDO (Fig. 2d) closely resembled that of pure Mg/Al-LDO (Fig. S3b), indicating that the oxide phase remained well dispersed after incorporation into the biochar matrix.^4,19^ Despite the collapse of the layered structure, the overall particle morphology was largely preserved, indicating that calcination altered the crystal structure without severely damaging the composite framework.^10,16,19,29^ This structural rearrangement may reduce the pore accessibility and specific surface area of the biochar/LDO composite.

#### 3.1.3. Textural properties

The textural properties of the prepared materials were evaluated by N_2_ adsorption/desorption isotherms at 77 K. Fig. S4 shows that all samples displayed type IV adsorption isotherms with H3 hysteresis loops, according to the IUPAC classification, indicating the presence of mesoporous structures.^34^ Some authors also reported a similar type IV isotherm and H3 hysteresis for spent coffee ground-derived^15^ and forestry waste-based biochar^13^ samples. The average pore diameters ranged from 7.71 to 15.0 nm, further confirming the mesoporous nature of all prepared materials (2.0–50 nm).

The *in situ* co-precipitation of Mg/Al-LDH on PF-biochar substantially decreased the *S*_BET_ (from 465.2 to 268.3 m^2^ g^−1^) and *V*_Total_ (from 1.745 to 0.622 cm^3^ g^−1^) values of the resultant composite of biochar/LDH when compared with PF-biochar (Table 1). This reduction is attributed to LDH precipitates, which partially blocked the external surface and the internal pore network of PF-biochar (Fig. 2b and c).^4,30^ These observations are consistent with the SEM images (Fig. 2), which show that the biochar surface became progressively covered by stacked LDH nanosheets following coprecipitation.

**Table 1:** Textural properties of PF-biochar, biochar/LDH, and biochar/LDO

	Unit	Prepared materials		
		PF-biochar	Biochar/LDH	Biochar/LDO
*S*_BET_	m^2^ g^−1^	465.2	268.3	241.2
*S*_External_	m^2^ g^−1^	451	259.7	239
*S*_Micropore_	m^2^ g^−1^	—	46.5	—
*V*_Total_	cm^3^ g^−1^	1.745	0.622	0.465
*V*_Micropore_	cm^3^ g^−1^	—	0.0209	—
Average pore diameter	nm	15	9.27	7.71

Biochar/LDH and biochar/LDO exhibited similar N_2_ adsorption/desorption isotherms (Fig. S5). The result suggests that calcination preserved the overall mesoporous structure of the composites. Nevertheless, *S*_BET_ and *V*_Total_ decreased slightly after calcination. This reduction is attributed to the partial collapse of the LDH layered structure, agglomeration of oxide particles (Fig. 2d), partial pore blockage, and structural rearrangement during the LDH-to-LDO transformation.^2,4,10,29^

#### 3.1.4. Surface functional groups

**Fig. 3:**
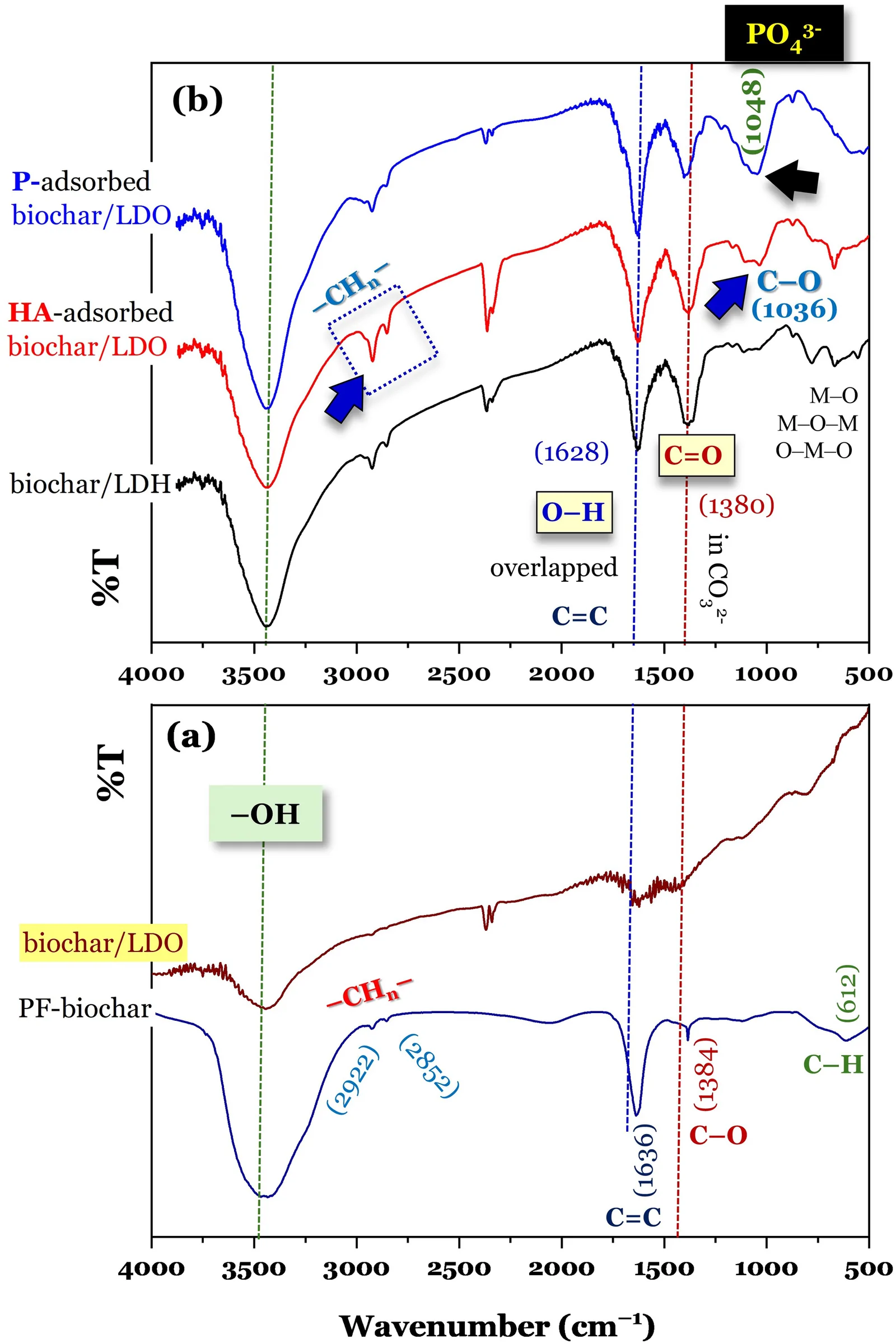
FTIR spectra of the (a) PF-biochar and biochar/LDO, and (b) biochar/LDH and biochar/LDO (after P and HA adsorption).

The surface functional groups of the PF-biochar, biochar/LDH, and biochar/LDO were characterized by FTIR spectroscopy (Fig. 3). The FTIR spectrum of PF-biochar (Fig. 3a) displayed several characteristic bands at approximately 3448 cm^−1^ (–OH group), 2922 and 2852 cm^−1^ (–CH_3_– and –CH_2_– stretching in aliphatic chains), 1636 cm^−1^ (C=C stretching vibration of aromatic structures), 1384 cm^−1^ (C–O stretching in phenolic or alcoholic groups), and 612 cm^−1^ (out-of-plane aromatic C–H bending).^13,15,21^ Notably, the C=O band was absent, whereas the C–O exhibited only weak intensity, indicating that most oxygen-containing functional groups had decomposed during pyrolysis. Similar observations have been reported for highly carbonised biochars.^13,30^ These results suggest that the oxygen-containing functional groups, particularly –COOH, were largely removed during pyrolysis at 600 °C, resulting in a highly carbonised biochar with enhanced aromaticity.

For biochar/LDH (Fig. 3b), the band at ∼3448 cm^−1^ is assigned to the –OH group from both PF-biochar and Mg/Al-LDH. The band near 1630 cm^−1^ results from the overlap of aromatic C=C vibrations from PF-biochar^19,30^ and the bending vibration of interlayer water in Mg/Al-LDH^13,25^ and other LDH types.^3^ A new pronounced band at approximately 1380 cm^−1^ is assigned to interlayer carbonate (CO_3_^2−^) species, confirming the successful formation of the LDH structure.^3,4,22,27^ Additional bands at approximately 535, 666, and 783 cm^−1^ correspond to M–O, M–O–M, and O–M–O vibrations (M = Mg^2+^ or Al^3+^), respectively.^11,27^

After calcination, the FTIR spectrum of biochar/LDO (Fig. 3a) showed a remarkable decrease in the intensity of the hydroxyl band at ∼3448 cm^−1^. Likewise, the bands associated with interlayer water (∼1620 cm^−1^) and carbonate (∼1380 cm^−1^) were significantly weakened or disappeared, indicating the removal of interlayer water and carbonate species during calcination.^2,27^ These spectral changes are consistent with dehydroxylation and decarbonation of the LDH layers during calcination, leading to the collapse of the layered structure and the formation of LDO.^4^ The disappearance of the characteristic hydroxyl and carbonate bands (Fig. 3a) further demonstrates the destruction of the original LDH structure and the formation of a metastable oxide phase, which is essential for subsequent structural reconstruction *via* the memory effect.^5,10,19,29^

These FTIR results are in excellent agreement with the XRD analysis, confirming the structural evolution of Mg/Al-LDH into Mg/Al-LDO while providing complementary evidence for the removal of interlayer species during calcination.

### 3.2. Adsorption study

#### 3.2.1. Effect of solution pH

**Fig. 4:**
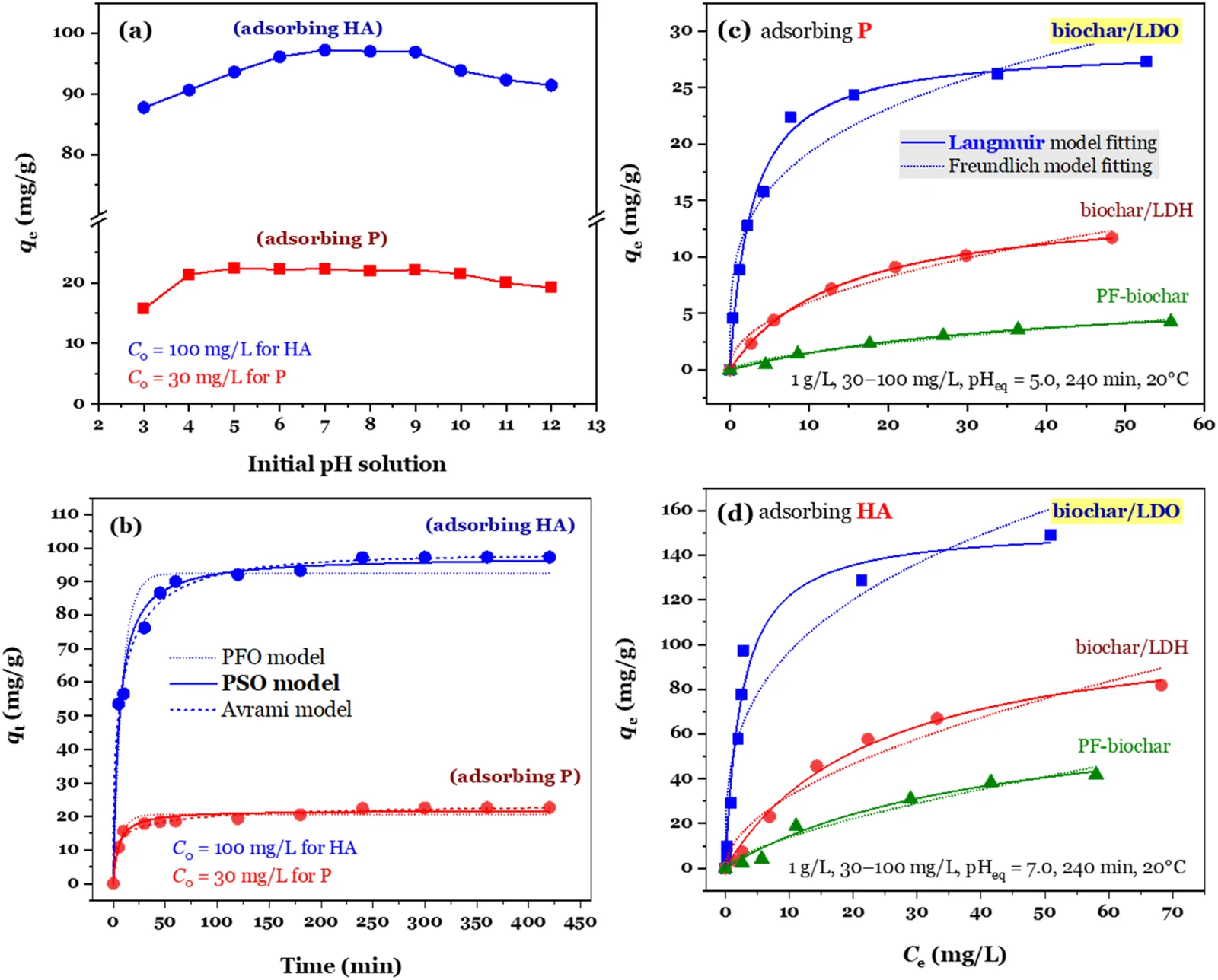
(a) and (b) Effect of solution pH and contact time on the adsorption process of P and HA onto biochar/LDO; and (c) and (d) the adsorption isotherm of P and HA onto PF-biochar, biochar/LDH, and biochar/LDO.

Solution pH is a key factor influencing adsorption because it governs both the adsorbent surface charge and the speciation of dissolved contaminants.^23^ As shown in Fig. 4a, the adsorption capacities of P and HA initially increased with increasing pH and then remained relatively stable over the pH range of 5–8 before gradually decreasing under more alkaline conditions. The highest adsorption capacity was 22.37 mg g^−1^ for phosphate at pH 5 and 97.24 mg g^−1^ for HA at pH 7. Specifically, the adsorption capacities increased from 15.71 to 22.37 mg g^−1^ for P (pH 3–5) and from 87.75 to 97.24 mg g^−1^ for HA (pH 3–7).

The point of zero charge (pH_PZC_) of biochar/LDO was 10.7 (Fig. S6), indicating that the biochar/LDO surface was positively charged at pH solution values below 10.7. Similar trends have been reported for phosphate adsorption by Zn/Al-LDH^3^ and HA adsorption by calcined magnetic Zn/Al-LDH.^16^ When the solution pH exceeded 8, the adsorption capacities gradually decreased by approximately 14% for phosphate and 5% for HA, consistent with previous studies by Zhang *et al.*^5^ and Khoshalhan Davoudli *et al.*,^35^ respectively.

The pH-dependent adsorption behaviour can be explained by changes in both the surface charge of biochar/LDO and the aqueous speciation of phosphate. Generally, H_2_PO_4_^−^ and HPO_4_^2−^ anions are the dominant phosphate species over the pH range pH 2–12 (Fig. S7).^2,5,28^ Because the adsorption free energy of H_2_PO_4_^−^ is lower than that of HPO_4_^2−^, the former is preferentially adsorbed.^5,19^ At pH values below the pH_PZC_ = 10.7, the biochar/LDO surface becomes positively charged owing to protonation of surface hydroxyl groups (−OH_2_^+^).^17,22^ These positively charged surface sites promote electrostatic attraction toward phosphate species (H_2_PO_4_^−^ and HPO_4_^2−^) and negatively charged HA molecules, thereby enhancing adsorption.^2,5,16^ Furthermore, the memory effect of LDO enables the reconstruction of the LDH structure under these conditions, facilitating the intercalation of phosphate anions into the regenerated interlayer galleries.^5,10^

In contrast, at pH values above the pH_PZC_, the deprotonation of hydroxyl groups on the material's surface resulted in a negatively charged surface, leading to electrostatic repulsion between the adsorbent and anionic phosphate species^23^ as well as HA anions.^12,16,35^ In addition, OH^−^ ions competed with phosphate or HA anions for active adsorption sites,^3,16^ thereby suppressing both electrostatic adsorption and anion intercalation.^10^ Therefore, pH 5 and pH 7 were selected as the optimum conditions for subsequent phosphate and HA adsorption experiments, respectively.

#### 3.2.2. Adsorption kinetics

Fig. 4b presents the time-dependent adsorption process for P and HA by biochar/LDO. The kinetic modelling results, adsorption rates, equilibrium times, and fitted parameters are summarised in Section S6 of the SI.

#### 3.2.3. Adsorption isotherm

The adsorption behaviour of P and HA on the PF-biochar, biochar/LDH, and biochar/LDO materials was evaluated using adsorption isotherm models (Fig. 4c and d). For all materials, the adsorption capacities increased with increasing initial concentration until equilibrium was reached. The fitted isotherm parameters (Table 2) showed that the Langmuir model provided a better fit (adj-*R*^2^ = 0.974–0.999) than the Freundlich model (adj-*R*^2^ = 0.878–0.977) for both P and HA.

**Table 2:** Isotherm models parameters for P and HA adsorption onto PF-biochar, biochar/LDH, and biochar/LDO

	Unit	PF-biochar		Biochar/LDH		Biochar/LDO	
		P	HA	P	HA	P	HA
**1. Langmuir model**							
*Q*_max_	mg g^−1^	7.40	74.1	15.0	112.7	28.6	152.9
*K*_L_	L mg^−1^	0.025	0.024	0.072	0.043	0.364	0.390
adj-*R*^2^	—	0.992	0.974	0.999	0.988	0.993	0.975
red-*χ*^2^	—	0.020	8.165	0.009	11.62	0.729	87.5

**2. Freundlich model**							
*K*_F_	(mg g^−1^)/(mg L^−1^)^*n*^	0.40	2.90	2.10	9.50	10.4	47.1
*n*	—	1.59	1.49	2.17	1.88	3.75	3.20
adj-*R*^2^	—	0.974	0.951	0.977	0.935	0.925	0.878
red-*χ*^2^	—	0.070	15.54	0.43	65.06	7.41	374

The maximum Langmuir adsorption capacities (*Q*_max_) of PF-biochar, biochar/LDH, and biochar/LDO for P were 7.4, 15.0, and 28.6 mg g^−1^, whereas the corresponding values for HA were 74.1, 112.7, and 152.9 mg g^−1^, respectively. The improvement in adsorption capacity followed the order PF-biochar < biochar/LDH < biochar/LDO, demonstrating that both LDH incorporation and subsequent calcination contributed synergistically to the development of high-performance adsorption sites. Similar improvements have been reported previously for phosphate adsorption by Mg/Al-LDH (64.10 mg g^−1^)^8^ and Mg/Al-LDO (101.83 mg g^−1^)^27^ as well as for HA adsorption by Zn/Al-LDH (84.0 mg g^−1^)^35^ and magnetic Zn/Al-LDO (165.8 mg g^−1^).^16^

Furthermore, Table S3 demonstrates that the *Q*_max_ of biochar/LDO compares favourably with those of previously reported adsorbents. Based on its superior adsorption performance, the biochar/LDO composite was selected as the adsorbent for all subsequent adsorption experiments.

#### 3.2.4. Adsorption thermodynamics

**Fig. 5:**
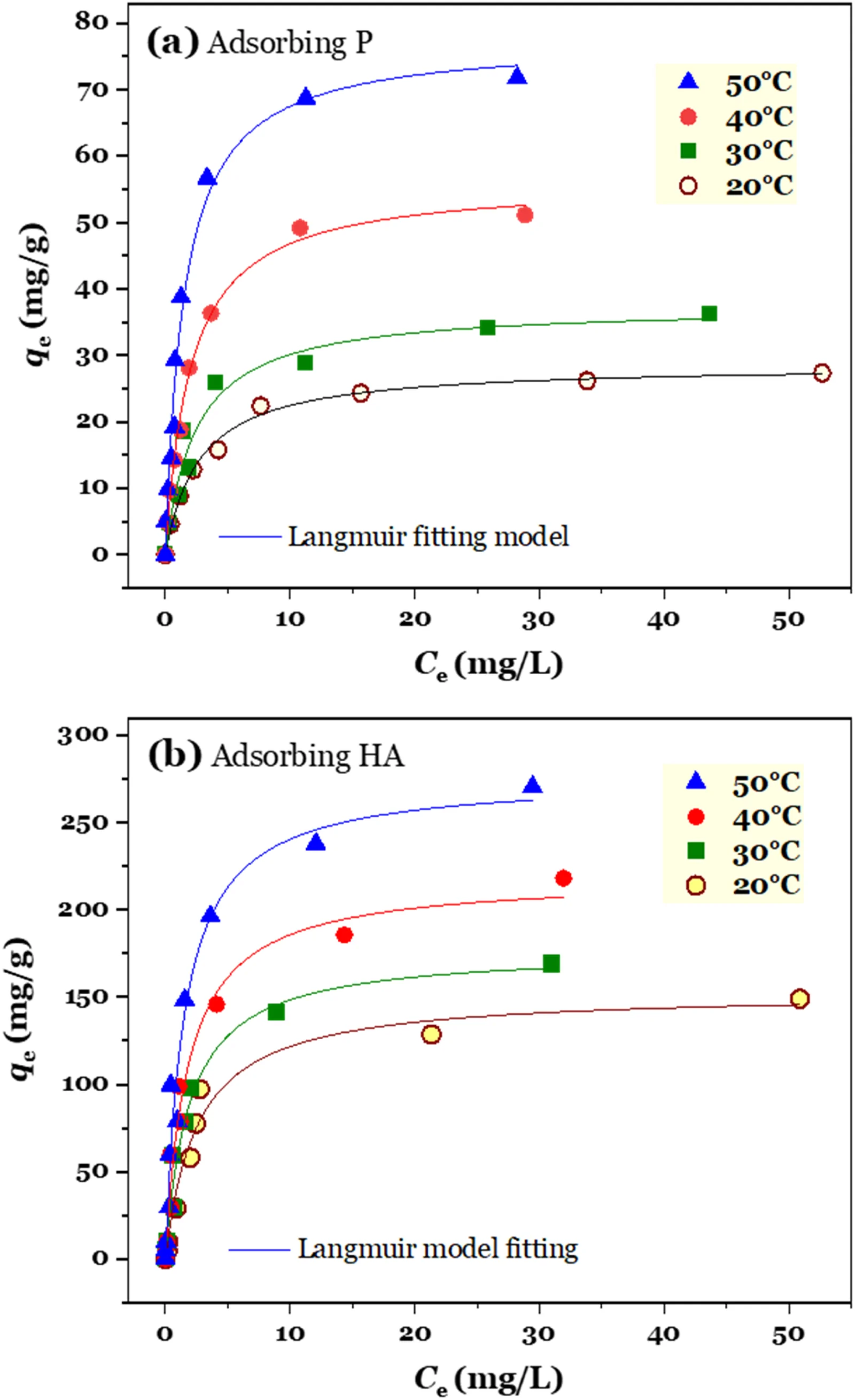
Adsorption isotherms of (a) P at pH = 5.0 and (b) HA at pH = 7.0 onto biochar/LDO at different temperatures.

Thermodynamic analysis was conducted to further elucidate the adsorption mechanisms of P and HA onto biochar/LDO. Adsorption experiments were performed at 293, 303, 313, and 323 K (Fig. 5). The change of the standard Gibbs energy (Δ*G*°), standard enthalpy (Δ*H*°), and standard entropy (Δ*S*°) were calculated based on the Langmuir isotherm using the equations provided in Section S7.

As summarised in Table S5, the negative Δ*G*° values indicate that phosphate adsorption onto biochar/LDO is thermodynamically favourable under the investigated conditions. In addition, the positive Δ*H*° values obtained for both P and HA indicate that the adsorption processes are endothermic, suggesting that higher temperatures favour adsorption. These results are consistent with previous studies on phosphate adsorption^2,4,7,18,27^ and HA adsorption.^13,16^ The relatively low Δ*H*° values (<80 kJ mol^−1^) suggest that physisorption was the predominant adsorption process.^36^ However, this does not exclude the contribution of other interactions as discussed in Section 3.5.

The positive Δ*S*° values indicate an increase in randomness at the solid–liquid interface during adsorption, reflecting enhanced freedom of the adsorbed species and favourable adsorption conditions.^2^

### 3.3. Reusability of biochar/LDO

**Fig. 6:**
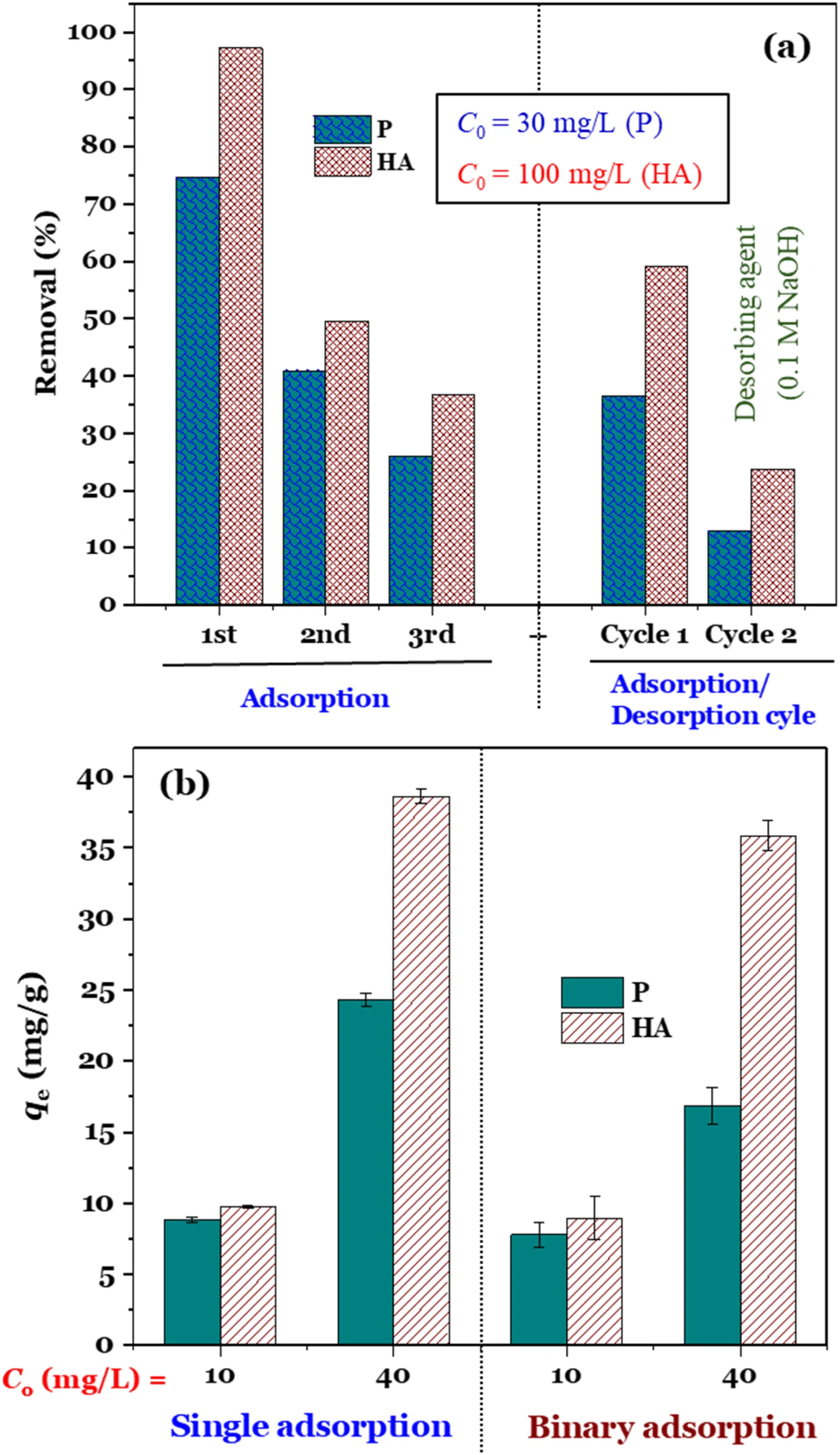
(a) Successive adsorption cycles followed by NaOH regeneration and re-adsorption of P and HA by biochar/LDO; and (b) single and binary systems (1 g L^−1^, pH_eq_ = 7, and 240 min).

The reusability of an adsorbent is a key factor in evaluating its practical applicability for sustainable and cost-effective water treatment. Fig. 6a shows that the removal efficiencies of P and HA gradually decreased from 75% and 97% in the first adsorption cycle to 26% and 37%, respectively, after the third cycle. The progressive decline in adsorption performance can be attributed to the gradual occupation of active adsorption sites by strongly adsorbed phosphate and HA molecules, incomplete desorption of adsorbed species, and partial pore blockage during repeated adsorption cycles.^4,17^

Following regeneration with 0.1 M NaOH, the recovery of adsorption performance reached 36% for P and 59% for HA, indicating that a substantial proportion of the active sites was regenerated. Regeneration with NaOH is attributed to the highly alkaline conditions, which increase the density of negatively charged surface groups and promote the desorption of adsorbed anionic species through electrostatic repulsion.^5^ In addition, hydroxide ions compete with the adsorbed phosphate and HA molecules for adsorption sites, thereby facilitating their release from the adsorbent surface.^4^

These results demonstrate that biochar/LDO possesses reasonable regeneration capability, although some irreversible adsorption or structural changes may contribute to a gradual decline in adsorption performance after repeated use.

### 3.4. Competitive and simultaneous adsorption of P and HA

The competitive adsorption behaviours of P and HA on the biochar/LDO composite under single- and binary-solute conditions are presented in Fig. 6b. When both phosphate and HA were present at 10 mg L^−1^, the adsorption capacities were nearly identical to those obtained under single-solute conditions, indicating that the available adsorption sites were sufficient to accommodate both species without significant competitive effects. When the HA concentration was increased to 40 mg L^−1^, phosphate adsorption capacity decreased by approximately 31%, demonstrating that HA effectively competed with phosphate for adsorption sites on biochar/LDO. The large molecular size of HA and its multiple oxygen-containing functional groups enable strong interactions with the adsorbent surface, thereby limiting the accessibility of phosphate to active adsorption sites.^6,14^ A similar competitive effect was reported by Lin *et al.*^14^ who observed that HA significantly suppressed phosphate adsorption onto Zr-modified zeolite because of competition for active binding sites. Likewise, Cheng *et al.*^6^ reported a 27% reduction in P adsorption by calcium aluminate decahydrate in the presence of 100 mg L^−1^ HA.

In contrast, our experimental results showed that coexistence with P at 40 mg L^−1^ had little influence on HA adsorption. This observation suggests that biochar/LDO exhibited a higher apparent affinity for HA than for phosphate under the investigated conditions, possibly because HA adsorption involves stronger, more favourable interactions with the adsorbent surface that are less susceptible to competitive inhibition by phosphate ions.

### 3.5. Mechanism exploration for P and HA adsorption onto biochar/LDO

Although biochar/LDO exhibited a lower specific surface area than PF-biochar, it showed a substantially higher adsorption capacity, indicating that adsorption performance was governed primarily by the chemical nature of the adsorption sites and the unique structural characteristics of LDO rather than surface area alone. To elucidate the adsorption mechanisms of P and HA, XRD and FTIR analyses were performed after adsorption.

Some LDO materials (*e.g.*, Zn/Al-LDO^16^ and Mg/Fe-LDO^28^) cannot reconstruct their original layered structures upon rehydration because their oxide structures remain stable after calcination. In general, the memory effect of LDO strongly depends on its metal composition and calcination conditions. In contrast, Fig. 1b shows that the characteristic layered structure of Mg/Al-LDH was regenerated after biochar/LDO was immersed in water, confirming the memory effect of Mg/Al-LDO.^28^ Similar reconstruction behaviour has been widely reported for Mg/Al-LDO systems.^4,8,18,27,31,32^ After rehydration in water, the reconstructed LDH exhibited a basal spacing of 0.741 nm (2*θ* = 11.9°), which was slightly smaller than that of the original biochar/LDH (0.755 nm, 2*θ* = 11.7°). This value is consistent with previously reported basal spacings of reconstructed Mg/Al-LDH.^10,28,31^ However, after phosphate and HA adsorption, the basal spacing increased to 0.882 nm and 1.147 nm, respectively, indicating expansion of the interlayer galleries resulting from the intercalation of phosphate and HA anions. The larger basal spacing observed after HA adsorption suggests greater interlayer expansion due to its larger molecular size compared with phosphate.

These results demonstrate that phosphate (predominantly H_2_PO_4_^−^ and HPO_4_^2−^) and HA anions were intercalated into the reconstructed LDH interlayers during rehydration, confirming that anion intercalation associated with the LDO memory effect was the dominant adsorption mechanism.^4,28,31^ Compared with pristine LDH, calcination transforms LDH into a metastable mixed oxide that readily reconstructs in the presence of water and suitable anions, accounting for the superior adsorption performance of LDO.^4,5,27,31^ The reconstruction of Mg/Al-LDO generally proceeds through a dissolution–rehydration–recrystallisation process, whereby the mixed oxides are progressively hydrated and regenerated into LDH in the presence of water and suitable anions.^10,29^

Calcination removes interlayer water, hydroxyl groups, and carbonate anions from LDH, generating coordinatively unsaturated metal sites, structural defects, and a higher density of exposed positively charged metal sites.^31,33^ These structural changes increase the affinity of LDO toward anionic contaminants and provide the driving force for structural reconstruction during rehydration.^4,27^ Unlike pristine LDH, whose interlayer galleries are initially occupied by carbonate anions, LDO reconstructs its layered structure in the presence of phosphate or humic acid, allowing these anions to be directly incorporated into the regenerated LDH interlayers through anion intercalation.^28^ During reconstruction, phosphate and HA anions restore charge neutrality by occupying the regenerated LDH interlayers, thereby simultaneously reconstructing the layered structure and enhancing the adsorption capacity of biochar/LDO.^28,31,33^

Although anion intercalation associated with the memory effect was identified as the dominant adsorption mechanism, it cannot account for the adsorption behaviour observed during the regeneration experiments alone. If anion intercalation associated with the memory effect were the only adsorption mechanism, the adsorption capacity would be expected to decrease substantially once the reconstructed LDH structure had been fully regenerated. However, Fig. 6a shows that biochar/LDO retained appreciable adsorption capacities for both phosphate and HA during the second and third adsorption cycles. These observations indicate that, in addition to anion intercalation, other adsorption mechanisms contributed to the removal of phosphate and HA.

The FTIR spectra further supported the proposed adsorption mechanism (Fig. 3b). After adsorption (P and HA), the FTIR spectrum of biochar/LDO closely resembled that of biochar/LDH, further supporting successful structural reconstruction. For phosphate-loaded biochar/LDO, a new band at approximately 1048 cm^−1^ was assigned to P–O stretching of adsorbed phosphate species.^4,8,9,18,27^ Likewise, HA-loaded biochar/LDO exhibited a new band at approximately 1036 cm^−1^, together with increased intensities of the bands near 2922 and 2852 cm^−1^, confirming the successful adsorption of HA.^16,37^

The relatively small shifts in the characteristic FTIR bands indicate that physical interactions played an important role during adsorption, although additional adsorption mechanisms also contributed.^9^ Overall, adsorption onto biochar/LDO was governed by multiple complementary mechanisms. Anion intercalation associated with the LDO memory effect was the dominant mechanism for both phosphate and HA. For phosphate, electrostatic attraction and surface complexation also contributed to adsorption.^4,9,15,27,28^ In contrast, HA adsorption additionally involved electrostatic attraction, hydrogen bonding between the –OH/–COOH groups of HA and surface hydroxyl groups of biochar/LDO,^13,16^ together with π–π interactions between the aromatic structures of HA and the carbon matrix of biochar.^15^

The combined evidence from XRD, FTIR, and regeneration experiments demonstrates that the superior adsorption performance of biochar/LDO originates from the synergistic contributions of memory-effect-driven anion intercalation and complementary surface interactions, rather than from an increase in specific surface area alone.

### 3.6. Estimation of preparation cost for biochar/LDO

Estimating the preparation cost of an adsorbent is important for assessing its potential practical applicability, as low-cost materials are generally more attractive for large-scale wastewater treatment. The direct operating cost (DOC) was applied in this study by considering only raw-material expenses and assuming that utility and labour costs contributed negligibly to the overall preparation cost.^38^

In this study, the DOC approach was adopted to estimate the preparation cost of 1 kg of biochar/LDO, including the costs of chemical reagents and the energy required for pyrolysis and drying. Palm fibre biomass was readily available, and its collection and transportation costs were assumed to be negligible under the present laboratory-scale conditions. The prices of the chemical reagents were obtained from Sigma-Aldrich and representative Chinese suppliers. Table S6 shows that the estimated DOC for preparing biochar/LDO was $1.86 per kg.

Although this estimate excludes capital investment, equipment depreciation, labour, and other indirect operating costs, it provides a reasonable preliminary assessment of the production cost at the laboratory scale. The low estimated preparation cost, together with the high adsorption capacity, good reusability, and the use of an abundant agricultural by-product as the carbon precursor, highlights the potential of biochar/LDO as a sustainable and economically attractive adsorbent for practical wastewater treatment applications.

## 4. Conclusions

A biochar/LDO composite was successfully synthesised by depositing Mg/Al-LDH onto PF-biochar, followed by calcination at 500 °C. SEM-EDS and XRD analyses confirmed the successful formation of Mg/Al-LDH on the biochar surface and its subsequent transformation into Mg/Al-LDO after calcination. Among the prepared materials, biochar/LDO exhibited the highest adsorption capacities for both phosphate (28.61 mg g^−1^) and humic acid (152.9 mg g^−1^), substantially outperforming biochar/LDH (15.0 and 112.7 mg g^−1^) and pristine PF-biochar (7.40 and 74.1 mg g^−1^), respectively.

Thermodynamic analysis demonstrated that the adsorption of phosphate (Δ*H*° = 15.92 kJ mol^−1^) and HA (Δ*H*° = 13.56 kJ mol^−1^) was endothermic and predominantly governed by physisorption, although multiple adsorption mechanisms were involved. XRD analysis demonstrated that anion intercalation associated with the LDO memory effect was the dominant adsorption mechanism. The expansion of the reconstructed LDH basal spacing from 0.741 nm to 0.882 nm after phosphate adsorption and to 1.147 nm after HA adsorption provided direct evidence for the intercalation of both anionic contaminants into the regenerated LDH interlayers. FTIR analysis, together with the adsorption, regeneration, and pH-dependent experiments, further demonstrated that phosphate adsorption involved electrostatic attraction and surface complexation, whereas HA adsorption additionally involved hydrogen bonding and π–π interactions. Binary adsorption experiments demonstrated that biochar/LDO maintained excellent adsorption performance under coexisting phosphate-HA conditions, providing a more realistic assessment of its behaviour in complex wastewater matrices than conventional single-solute systems.

The estimated direct operating cost for preparing biochar/LDO was approximately $1.86 per kg. Overall, the superior adsorption performance of biochar/LDO is attributable to the synergistic contribution of memory-effect-driven anion intercalation and complementary surface interactions, rather than to a high specific surface area. In addition to its low preparation cost, satisfactory reusability, and preparation from abundant agricultural biomass, biochar/LDO represents a promising and sustainable adsorbent for the simultaneous removal of inorganic and organic anionic contaminants from wastewater.

## Author contributions

Racha Kara: conceptualisation, data curation, investigation, resources, formal analysis, writing – original draft. Abdelkader Ouakouak: conceptualisation, supervision, funding acquisition, investigation, validation, writing – review and editing. Lynda Hecini: writing – review and editing. Dhirar Ben Salem: investigation and writing – review and editing. Noureddine Rouahna: data curation, investigation. Fouzia Touahra: writing – review and editing. Nesrine Zenidi: software, review & editing. David Houivet: writing – review and editing. Nguyen Trong Anh: resources, writing – review and editing. Hai Nguyen Tran: conceptualisation, validation, formal analysis, visualization, software, writing – review and editing.

## Conflicts of interest

The authors declare that they have no known competing financial interests or personal relationships that could have appeared to influence the work reported in this paper.

## Supplementary Material

RA-016-D6RA03448A-s001

## Data Availability

The data supporting the findings of this study are available within the article and its supplementary information (SI). Additional data, including raw experimental measurements, adsorption data, and the data on N_2_ adsorption/desorption isotherms at 77 K, FTIR and XRD, are available from the corresponding author upon reasonable request. Supplementary information is available. See DOI: https://doi.org/10.1039/d6ra03448a.
