# Supplementary material for: Hybrid biochar/layered double oxide for phosphate and humic acid removal from water: dominant role of memory-effect-driven anion intercalation
Source: RSC Adv. 2026 Aug 20;16(41):49100–11. doi: 10.1039/d6ra03448a (PMC13491316; doi:10.1039/d6ra03448a)
Supplement: Supplementary file 1 [file RA-016-D6RA03448A-s001.pdf]

Supplementary Information (SI) for RSC Advances.
This journal is © The Royal Society of Chemistry 2026

SUPPORTING INFORMATION

Section S1. Reagents and materials

Sodium phosphate monobasic monohydrate ($NaH_2PO_4 \cdot H_2O$) was purchased from J.T.Baker (Inc., Philipsburg). Magnesium chloride hexahydrate ($MgCl_2 \cdot 6H_2O$), aluminium chloride hexahydrate ($AlCl_3 \cdot 6H_2O$), sodium chloride (NaCl), sodium carbonate (Na_2CO_3), sodium hydroxide (NaOH), hydrochloric acid (HCl), and humic acid were obtained from Sigma-Aldrich (St. Louis, USA). Potassium antimony oxide tartrate hemihydrate ($C_4H_4KO_7SB.0.5H_2O$) and sulfuric acid (H_2SO_4) were sourced from Biochem Chemopharma (Quebec, Canada), while ammonium heptamolybdate tetrahydrate ($(NH_4)_6MO_7O_{24} \cdot 4H_2O$) and ascorbic acid were procured from VWR Chemicals (Radnor, Pennsylvania, USA). All reagents were of analytical grade, and all experimental solutions were prepared using distilled water.

Section S2. Materials' characterization

The physical properties of the prepared material were characterized using the following techniques. The crystallinity of the samples was determined using X-ray diffraction (XRD; Bruker D8 Advance) with Cu Kα1 radiation (λ = 1.54 Å). The experimental conditions were set to a voltage of 33 kV, a current of 45 mA, and a 2θ range of 5° to 65° with a step size of 0.02°.

The surface functional groups were analysed by Fourier transform infrared spectroscopy (FTIR, Cary 630, Agilent Technologies) over the 400-4000 cm^{-1} range. The resolution was ≤ 2 cm^{-1}, and the device's wavenumber accuracy was 0.05 cm^{-1}.

A scanning electron microscope (SEM; Apreo 2C, Thermo Fisher Scientific) coupled with energy-dispersive spectroscopy (EDS) was employed to analyse the surface morphology and elemental composition of the adsorbents.

The specific surface area and porosity were determined using N_2 adsorption/desorption isotherms at 77 K, measured with a Micromeritics ASAP 2020 instrument based on the conventional methods (BET and BJH).

The pH drift method was used to determine the point of zero charge (pH_{PZC}). Briefly, a series of 50 mL NaCl (0.1 M) solutions was poured into flasks, with pH values ranging from 2 to 12, and the initial pH (pH_i) was recorded. Next, 50 mg of adsorbent was added to each solution and stirred at room temperature for 4 h. The mixture was filtered to separate the adsorbent, then the final pH (pH_f) of the filtrate was measured using an accurate pH meter. The pH_{PZC} value (where ΔpH = 0) was obtained from a plot of (pH_f - pH_i) as a function of pH_i. This procedure has been detailed in previous studies (Salem et al., 2023).

Section S3. Analysis of adsorption data

The amount of P or HA adsorbed per unit mass of the material at a given time t (q_t, mg/g) and at equilibrium (q_e, mg/g) was calculated using Eq. (S1) and (S2).

$$q_t = \frac{C_0 - C_t}{m} \times V \quad \text{(S1)}$$

$$q_e = \frac{C_0 - C_e}{m} \times V \quad \text{(S2)}$$

where C_t (mg/L) represents the concentration of P or HA at any time t during the adsorption process; V (L) is the total volume of the solution; and m (g) is the mass of the material used for adsorption.

To model the adsorption kinetics of P and HA, three widely used mathematical models were applied: the pseudo-first-order (PFO) and pseudo-second-order (PSO) models (Tran et al., 2017) and the Avrami model (George & Sugunan, 2014). The Langmuir and the Freundlich isotherm models were employed to interpret and fit the equilibrium adsorption data (Lima et al., 2015). The equations and parameters of the models used are presented in Table S2.

The selection of the most appropriate kinetic or isotherm model was based primarily on the adjusted coefficient of determination (adj-R^2) and the reduced chi-square statistic (red-χ^2) (Tran et al., 2017) (Eq. (S4) and (S5)). These parameters were calculated using Origin software. A higher adj-R^2 and a lower red-χ^2 indicate a better statistical fit to the experimental data.

$$R^2 = 1 - \frac{\sum (q_{e,exp} - q_{e,cal})^2}{\sum (q_{e,exp} - q_{e,mean})^2} \quad \text{(S3)}$$

$$adj - R^2 = 1 - \left[(1 - R^2)(\frac{n-1}{n-p})\right] \quad \text{(S4)}$$

$$red - \chi^2 = \frac{\sum (q_{e,exp} - q_{e,cal})^2}{n - p} \quad \text{(S5)}$$

where $q_{e,exp}$ represents the experimental equilibrium adsorption capacity; $q_{e,cal}$ corresponds to the adsorption capacity predicted by the model; $q_{e,mean}$ denotes the mean of the $q_{e,exp}$ values; n is the number of experimental data points; and p is the number of fitted parameters in the model.

Section S4. Hydrotalcite

Allmann R, Jepsen H P
Neues Jahrbuch fur Mineralogie, Monatshefte 1969 (1969) 544-551
Die struktur des hydrotalkits
Locality: Vezna, Western Moravia
_database_code_amcsd 0014738

CELL PARAMETERS: 3.0540 3.0540 22.8100 90.000 90.000 120.000
SPACE GROUP: R-3m
X-RAY WAVELENGTH: 1.541838
Cell Volume: 184.244
Density (g/cm3): 2.144
MAX. ABS. INTENSITY / VOLUME**2: 15.82458594
RIR: 2.403
RIR based on corundum from Acta Crystallographica A38 (1982) 733-739

2-THETA	INTENSITY	D-SPACING	H K L	Multiplicity
11.64	100.00	7.6033	0 0 3	2
23.40	34.54	3.8017	0 0 6	2
34.13	2.25	2.6272	1 0 1	6
34.82	30.60	2.5765	0 1 2	6
35.42	1.89	2.5344	0 0 9	2
39.38	25.32	2.2881	0 1 5	6
46.85	30.73	1.9391	0 1 8	6
53.01	7.34	1.7273	1 0 10	6
56.38	4.76	1.6319	0 1 11	6
60.64	9.67	1.5270	1 1 0	6
61.99	9.24	1.4971	1 1 3	12
63.64	4.12	1.4621	1 0 13	6
65.92	3.00	1.4170	1 1 6	12
67.52	1.49	1.3872	0 1 14	6
71.87	1.18	1.3136	2 0 2	6
74.74	2.66	1.2701	2 0 5	6
75.80	3.41	1.2549	1 0 16	6
79.97	2.37	1.1997	2 0 8	6
87.48	1.12	1.1150	2 0 11	6

===

XPOW Copyright 1993 Bob Downs, Ranjini Swaminathan and Kurt Bartelmehs

For reference, see Downs et al. (1993) American Mineralogist 78, 1104-1107.

https://www.rruff.net/odr/amcsd#/odr/search/display/0/eyI3MTk3IjoiXCJIeWRyb3RhbGNpdGVcIiIsImR0X2lkIjoiNzcxIn0/1

Section S5. Periclase

Hazen R M

American Mineralogist 61 (1976) 266-271

Effects of temperature and pressure on the cell dimension and X-ray temperature factors of periclase

T = 24 C, P = 1 atm, standard mount

_database_code_amcsd 0000501

CELL PARAMETERS: 4.2110 4.2110 4.2110 90.000 90.000 90.000

SPACE GROUP: Fm3m

X-RAY WAVELENGTH: 1.541838

Cell Volume: 74.672

Density (g/cm3): 3.585

MAX. ABS. INTENSITY / VOLUME**2: 35.75123052

RIR: 3.247

RIR based on corundum from Acta Crystallographica A38 (1982) 733-739

2-THETA	INTENSITY	D-SPACING	H	K	L	Multiplicity
36.97	10.52	2.4312	1	1	1	8
42.96	100.00	2.1055	2	0	0	6
62.37	53.43	1.4888	2	2	0	12
74.77	6.56	1.2697	3	1	1	24
78.72	15.30	1.2156	2	2	2	8

==

XPOW Copyright 1993 Bob Downs, Ranjini Swaminathan and Kurt Bartelmehs

For reference, see Downs et al. (1993) American Mineralogist 78, 1104-1107.

https://www.rruff.net/odr/amcsd#/odr/search/display/0/eyI3MTk3IjoiXCJQZXJpY2xhc2VcIiIsImR0X2lkIjoiNzcxIn0/1

Section S6. Adsorption kinetics

Kinetic experiments were conducted to evaluate P and HA biochar/LDO adsorption as a function of contact time (Fig. 4b). A steep increase in adsorption for P and HA occurred during the first 2 h of contact, representing over 86% of the total adsorption achieved at equilibrium. This trend is attributed to the numerous active sites in the adsorbent, which favour the adsorption (Bezza et al., 2024). After this, the system approached equilibrium, with most available adsorption sites saturated, and P and HA adsorption reached equilibrium in 240 minutes, yielding adsorption capacities of 22.37 mg/g and 97.24 mg/g, respectively. The PFO, PSO, and Avrami kinetic models were used to fit the experimental data, with the corresponding fitting parameters presented in Table S4. The adsorption of P and HA onto biochar/LDO was better described by the PSO and Avrami models than by the PFO model, with the highest coefficients of determination ranging from 0.987 to 0.992. Moreover, the theoretical values predicted by the PSO model were in close agreement with the experimental adsorption capacities, suggesting that this model is more appropriate for describing the experimental data.

Section S7. Thermodynamic Equations

The thermodynamic parameters were calculated using the following equations:

$$\Delta G^0 = -RT \ln K_C \tag{S6}$$

The relationship among $\Delta H°$, $\Delta G°$ and $\Delta S°$ is expressed as follows:

$$\Delta G^0 = \Delta H^0 - T\Delta S^0 \tag{S7}$$

The van't Hoff relation is obtained by substituting Eq. (S6) into Eq. (S7)

$$\ln K_C = \frac{-\Delta H^0}{R} \times \frac{1}{T} + \frac{\Delta S^0}{R} \tag{S8}$$

where T is the temperature in kelvin (K), and R is the universal gas constant (8.31446 J/(mol × K)).

The thermodynamic equilibrium constant K_C was calculated using Eq. (S9):

$$K_C = \frac{K_L}{\gamma_{Adsorbate}} C^0 \tag{S9}$$

where K_L (L/mol) is the constant of the Langmuir model; C^0 (mol/L) is the standard concentration of adsorbate (1 mol/L); $\gamma_{adsorbate}$ is the activity coefficient of adsorbate, which is unitless and approaches 1 under dilute conditions (Tran et al, 2022). In this study, the M_w of P was taken as 96.97 g/mol, while that of HA is undefined. Therefore, for HA, only $\Delta H°$ was considered in the discussion (as it is not affected by the unit of K_L).

The values of $\Delta H°$ (kJ/mol) and $\Delta S°$ (J/(mol×K)) are calculated from the slope and intercept, respectively, of the line obtained by plotting ln Kc as a function of 1/T; $\Delta G°$ (KJ/mol) is determined directly from Eq. (S7) (Tran et al., 2017).

Table S1. Summary of experimental conditions for the adsorption of P and HA onto biochar/LDO composite

Operating conditions	Concentration C_0 (mg/L)		Time t (min)		Solution pH		Temperature T (°C)	
	**P**	**HA**	**P**	**HA**	**P**	**HA**	**P**	**HA**
Effect of initial concentration	5-80	5-200	240	240	5	7	20	20
Effect of contact time	30	100	5-420	5-420	5	7	20	20
Effect of solution pH	30	100	240	240	3-12	3-12	20	20
Effect of temperature	30	100	240	240	5	7	20-50	20-50
Binary system	10 and 40	10 and 40	240	240	7	7	20	20

Note: The pH was adjusted with 0.1 M NaOH or 0.1 M HCl

Table S2. Description of kinetic and isothermal adsorption models: equations and parameters

Models	Equations	Parameters
**Kinetic models**		
1. Pseudo-first-order (PFO)	$q_t = q_e \times (1 - e^{-k_1 t})$	q_t and q_e (mg/g) represent the amounts of P and HA adsorbed at time t and at equilibrium, respectively.
2. Pseudo-second-order (PSO)	$q_t = \frac{q_e^2 \times k_2 \times t}{1 + q_e \times k_2 \times t}$	k_1 (1/min) is the rate constant of the PFO.
		k_2 (g/(min × mg)) is the rate constant of the PSO.
3. Avrami	$q_t = q_e \times (1 - e^{-(k_{av} \times t)^{n_{av}}})$	k_A (1/min) is the Avrami kinetic constant.
		n_{av} is an exponent that is dependent on the adsorption mechanism.
**Isotherm models**		
1. Langmuir	$q_e = \frac{Q_{max} \times K_L \times C_e}{1 + K_L \times C_e}$	Q_{max} (mg/g) is the maximum adsorption capacity of the Langmuir model.
2. Freundlich	$q_e = K_F \times C_e^{\frac{1}{n}}$	C_e (mg/L) is the concentration of P and HA at equilibrium.
		K_L (L/mg) is the constant of Langmuir.
		K_F [(mg/g)/(mg/L)^n] is the constant of the Freundlich model.
		n (dimensionless) is the parameter of the Freundlich intensity.

Table S3. Comparison of P and HA adsorption capacities on biochar/LDO with those of other reported adsorbents

Pollutant	Adsorbents	Adsorption capacity (mg/g)	*T* (°C)	Time (h)	References
**P**	Biochar doped with $MgCl_2$	17.6	—	24	Novais et al., 2018
	Zr/Al-pillared montmorillonite	17.2	25	6	Huang et al., 2015
	Chitosan-Calcite adsorbent	21.36	22	2	Pap et al., 2020
	Biochar $Mg(OH)_2$/bentonite composite	34.6	25	4	Jing et al., 2019
	Al extract impregnated Korean pine residue biochar	11.56	RT	48	Van et al., 2023
	$AlCl_3$ impregnated biochar	2.26			
	Mg-Fe LDHs-loaded biochar	17.46	RT	1	Bolbol et al., 2019
	**Biochar/LDO**	**28.61**	**20**	**4**	**This study**
**HA**	Bamboo biochar	5.15	20	48	Yan et al., 2020
	MOP-functionalized biochar	87.1	25	2.5	Belbali et al., 2022
	Graphitic Carbon Nitride	73.24	25	2.5	Yue et al., 2021
	Modified aged refuse	37.31	25	1	Zhang et al., 2018
	Activated carbon modified by multi-wall carbon nanotubes	43.29	RT	0.5	Noorimotlagh et al., 2020
	Granular-activated carbon (GAC)	34.6	RT	2 h	Mahfoudhi et al., 2022
	Activated carbon coated with nZVI nanoparticles (AC-nZVI)	86.95	—	45 min	Rashtbari et al., 2022
	Flake Zn-Al layered double hydroxides	84	25	2	Davoudli et al., 2021
	Microspheres of LDH (MLDHs)	59.9		280 min	
	**Biochar/LDO**	**152.93**	**20**	**4 h**	**This study**

Note: RT is the room temperature.

Table S4. Parameters of kinetic models for P and HA adsorption onto biochar/LDO.

	**P**	**HA**
$q_{e,\ experiment}$ **(mg/g)**	22.37	97.24
**1. Pseudo-first-order (PFO)**		
q_e (mg/g)	20.67	92.45
k_1 (1/min)	0.109	0.116
adj-R^2	0.922	0.939
red-χ^2	3.330	52.0
**2. Pseudo-second-order (PSO)**		
q_e (mg/g)	21.97	97.5
k_2 (g/(min × mg))	0.007	0.002
adj-R^2	0.978	0.983
red-χ^2	1.017	13.99
**3. Avrami**		
q_e (mg/g)	23.35	97.94
k_{av} (1/min)	0.070	0.095
n_{av}	0.356	0.455
adj-R^2	0.987	0.992
red-χ^2	0.543	6.737

Table S5. Thermodynamic parameters for P and HA adsorption onto biochar/LDO.

Adsorbate	T (K)	K_L (L/mg)	K_C	van't Hoff equation	Thermodynamic parameters		
					$\Delta G°$ (kJ/mol)	$\Delta H°$ (kJ/mol)	$\Delta S°$ [J/(mol × K)]
P	293	0.3641	35,307	$y = -1915x + 16.96$	–25.51	15.92	140.99
	303	0.4109	39,845	$r^2 = 0.947$	–26.69		
	313	0.4956	48,058		–28.05		
	323	0.6744	65,397		–29.78		
HA	293	0.3901		$y = -1631x + 4.66$	—	13.56	—
	303	0.5193		$r^2 = 0.958$			
	313	0.5611					
	323	0.6734					

Table S6. Cost estimated for the synthesis of one kg of biochar/LDO composite

Chemicals used	Amount used (kg)	Price (US$/kg)	Total (US$/kg)
$MgCl_2·6H_2O$	1.316	0.08	0.105
$AlCl_3.6H_2O$	0.780	1.013	0.790
Na_2CO_3	1.039	0.29	0.301
NaOH	0.784	0.40	0.314
		Total	1.509
	Total direct operating cost (DOC) = 1.509 + 0.35 = 1.86 US$/kg		

Note: The energy price per kWh was estimated at 0.03 US$, based on the Algerian government's official rate. The total energy cost (kWh) to produce 1 kg of materials was approximately 0.35.

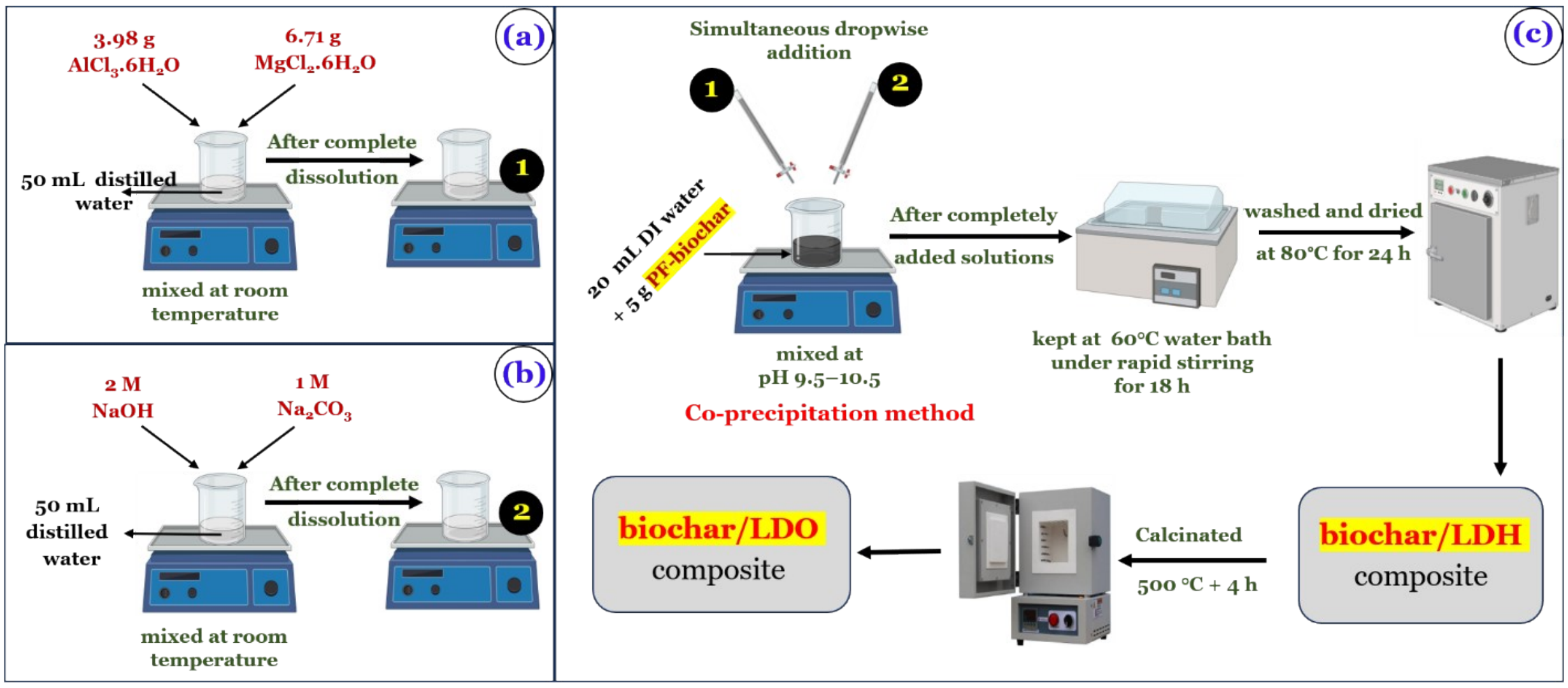

Figure S1. Experimental procedure for the preparation **(a)** Solution 1, **(b)** Solution 2, and **(c)** the composites of biochar/LDH and biochar/LDO

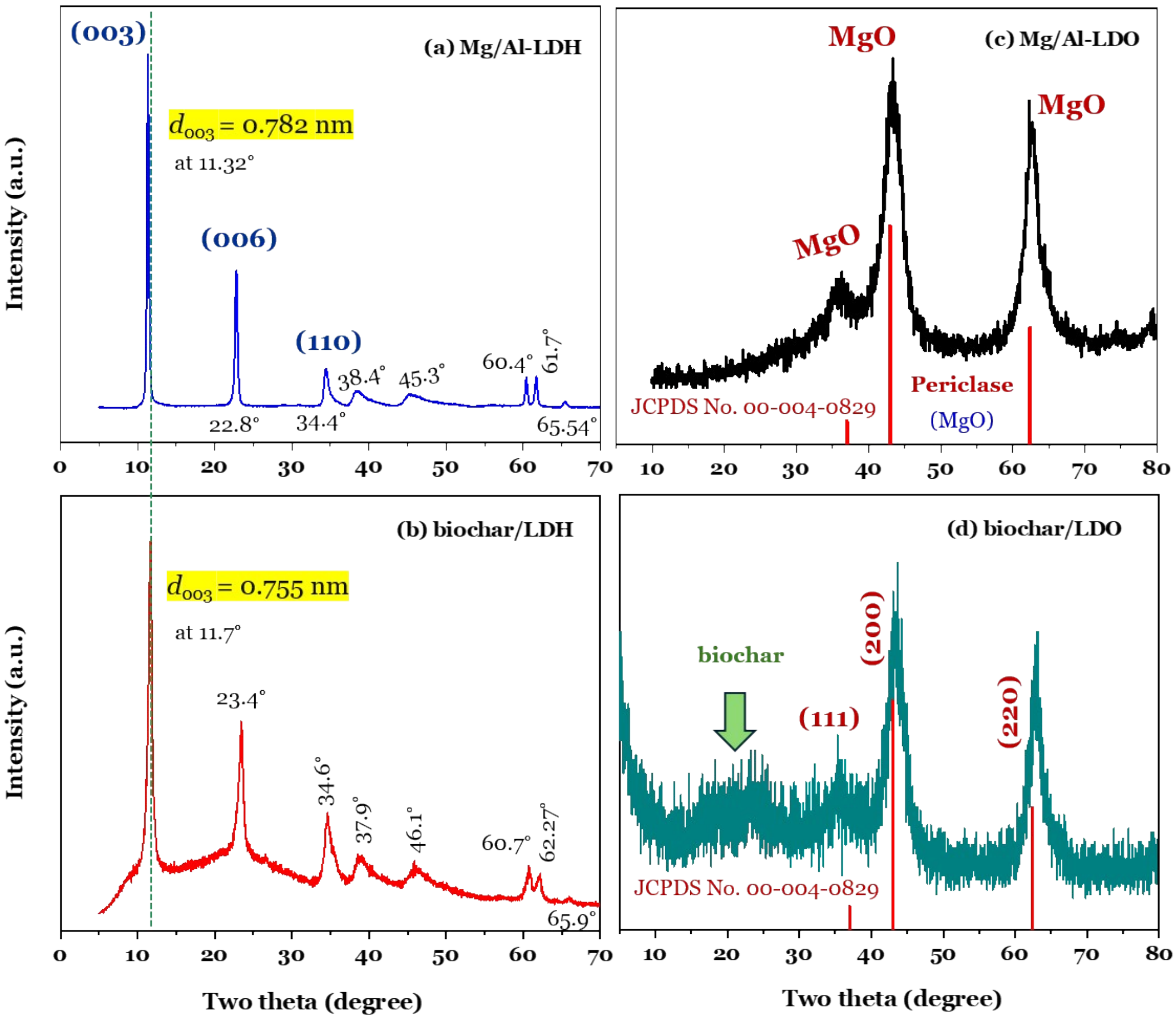

Figure S2. XRD spectrum of **(a)** Mg/Al-LDH, **(b)** biochar/LDH, **(c)** Mg/Al-LDO, and **(d)** biochar/LDO

(a)

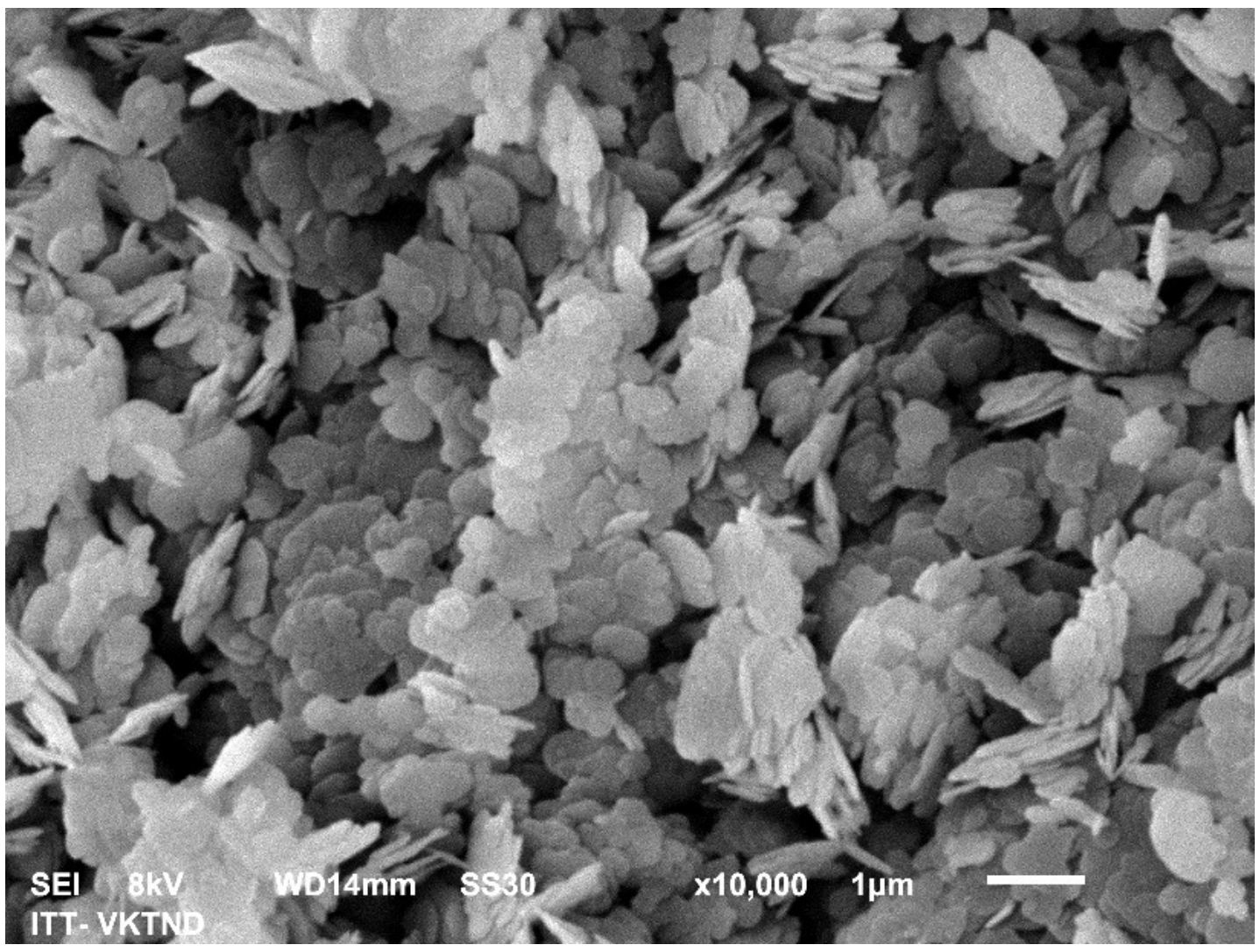

(b)

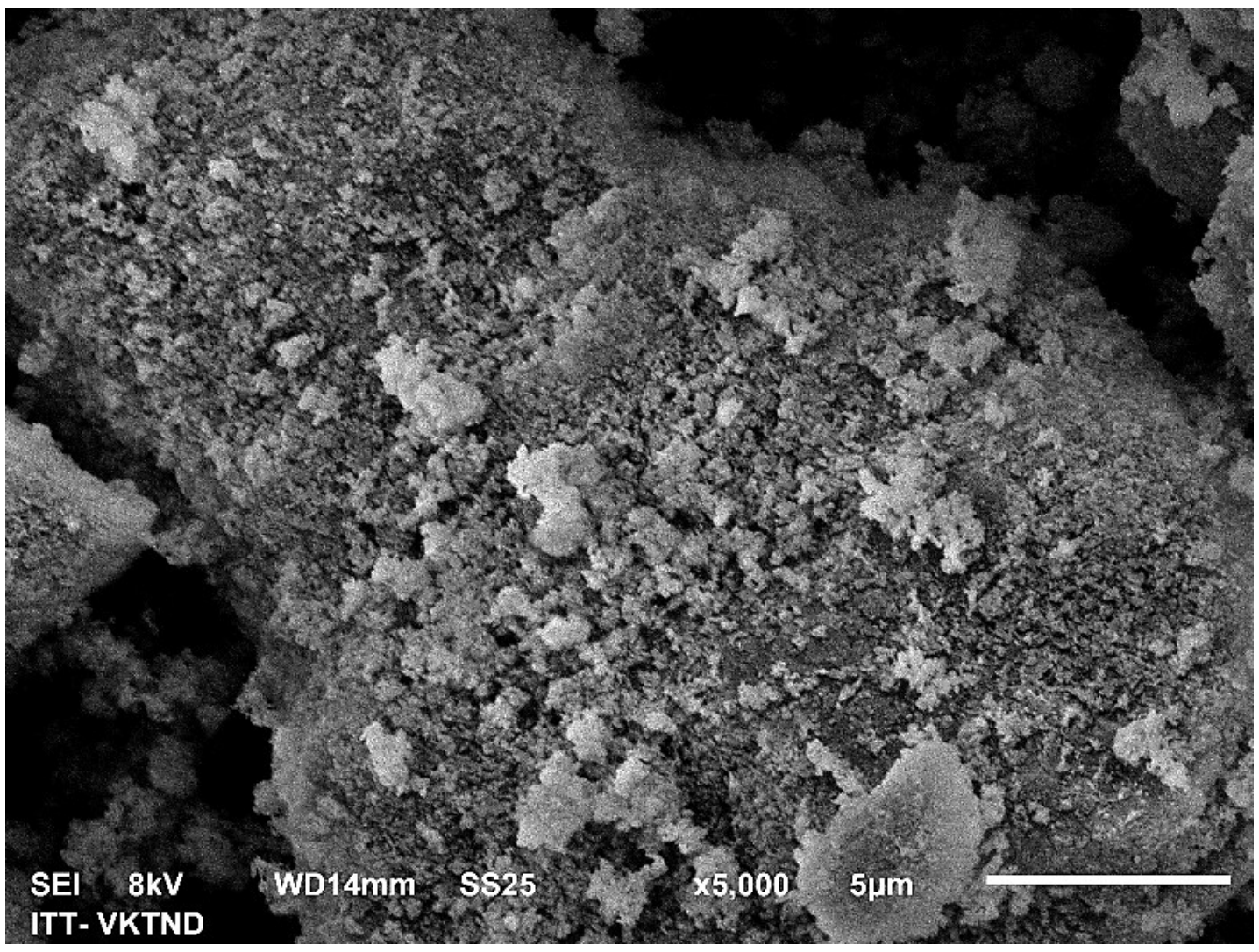

Figure S3. SEM images of **(a)** pure Mg/Al-LDH and **(b)** pure Mg/Al-LDO reference materials (the data detected by JSM-6510 LV)

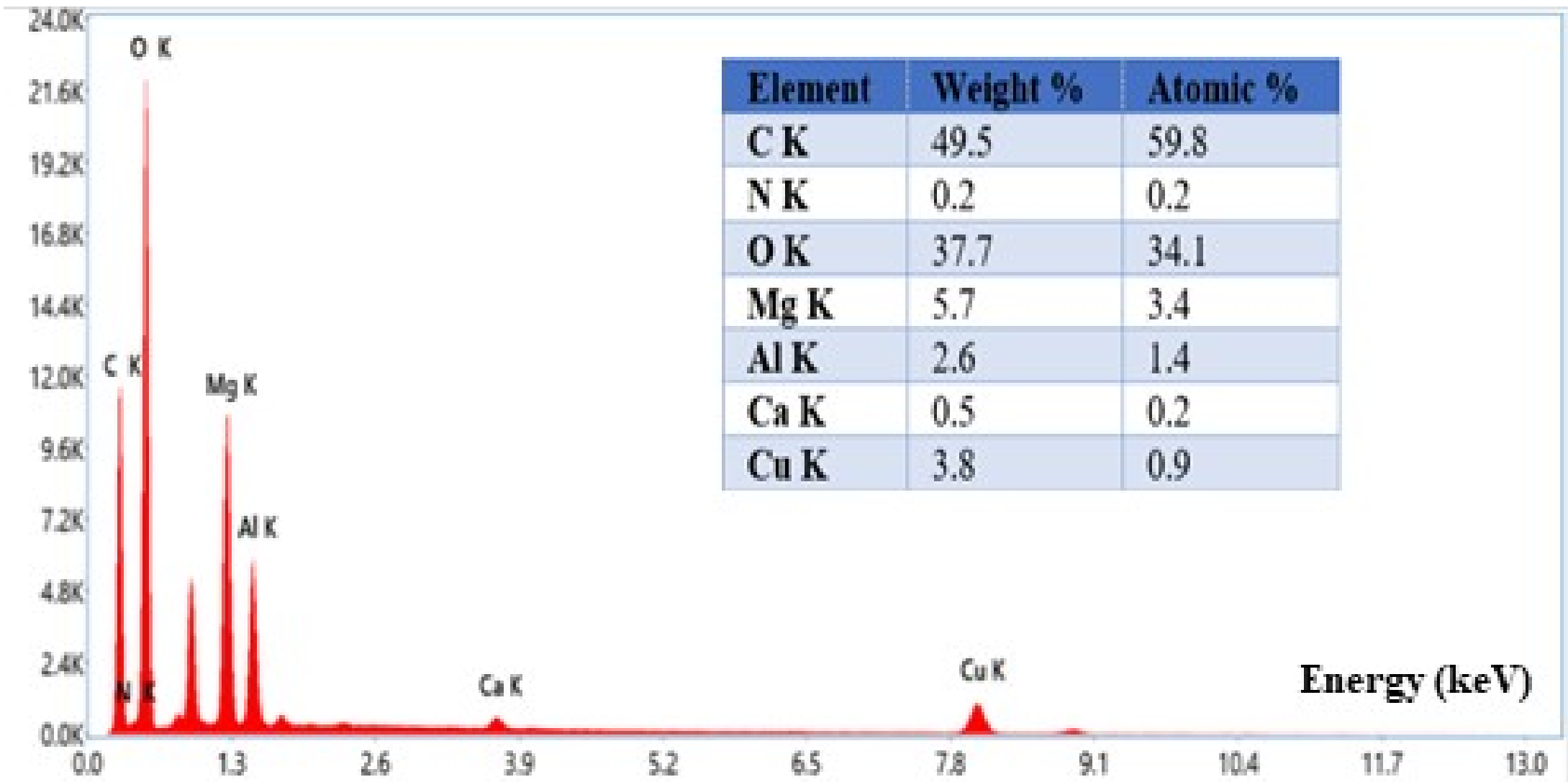

Element	Weight %	Atomic %
C K	49.5	59.8
N K	0.2	0.2
O K	37.7	34.1
Mg K	5.7	3.4
Al K	2.6	1.4
Ca K	0.5	0.2
Cu K	3.8	0.9

Figure S4. EDS spectrum of biochar/LDH

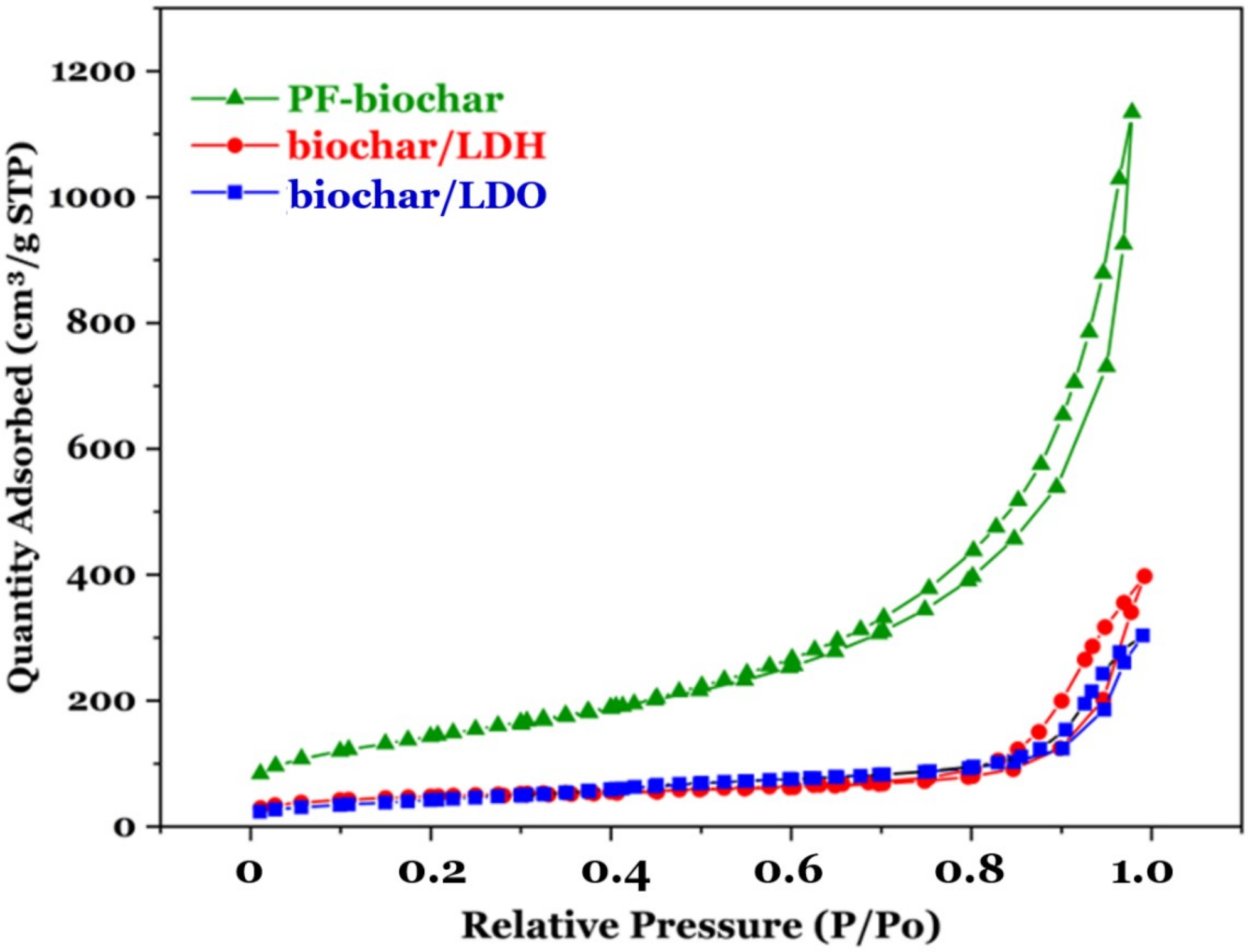

Figure S5. N_2 adsorption/desorption isotherms at 77 K of PF-biochar, biochar/LDH composite, and biochar/LDO composite

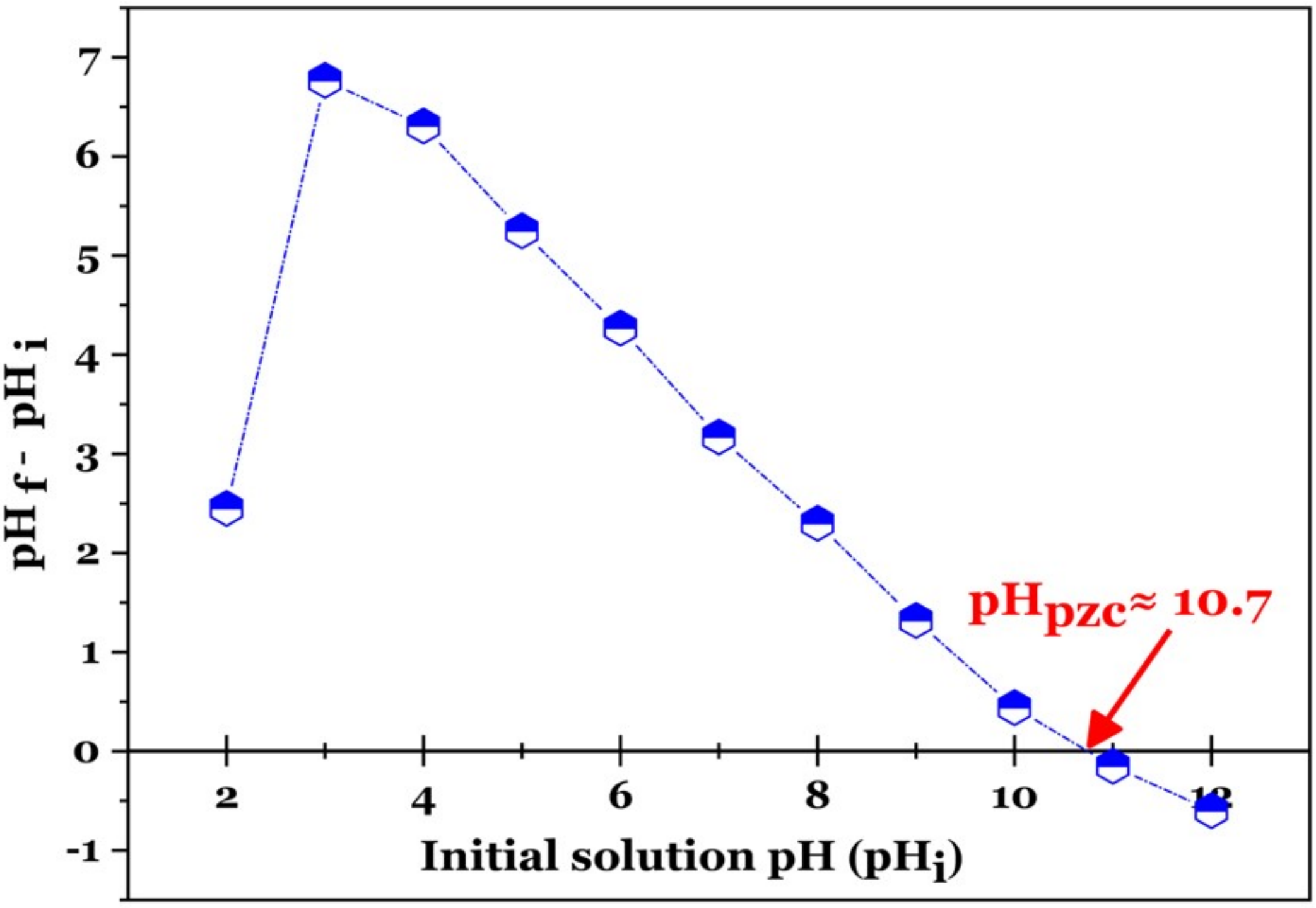

Figure S6. pH_{PZC} value of biochar/LDO composite found by the drift method

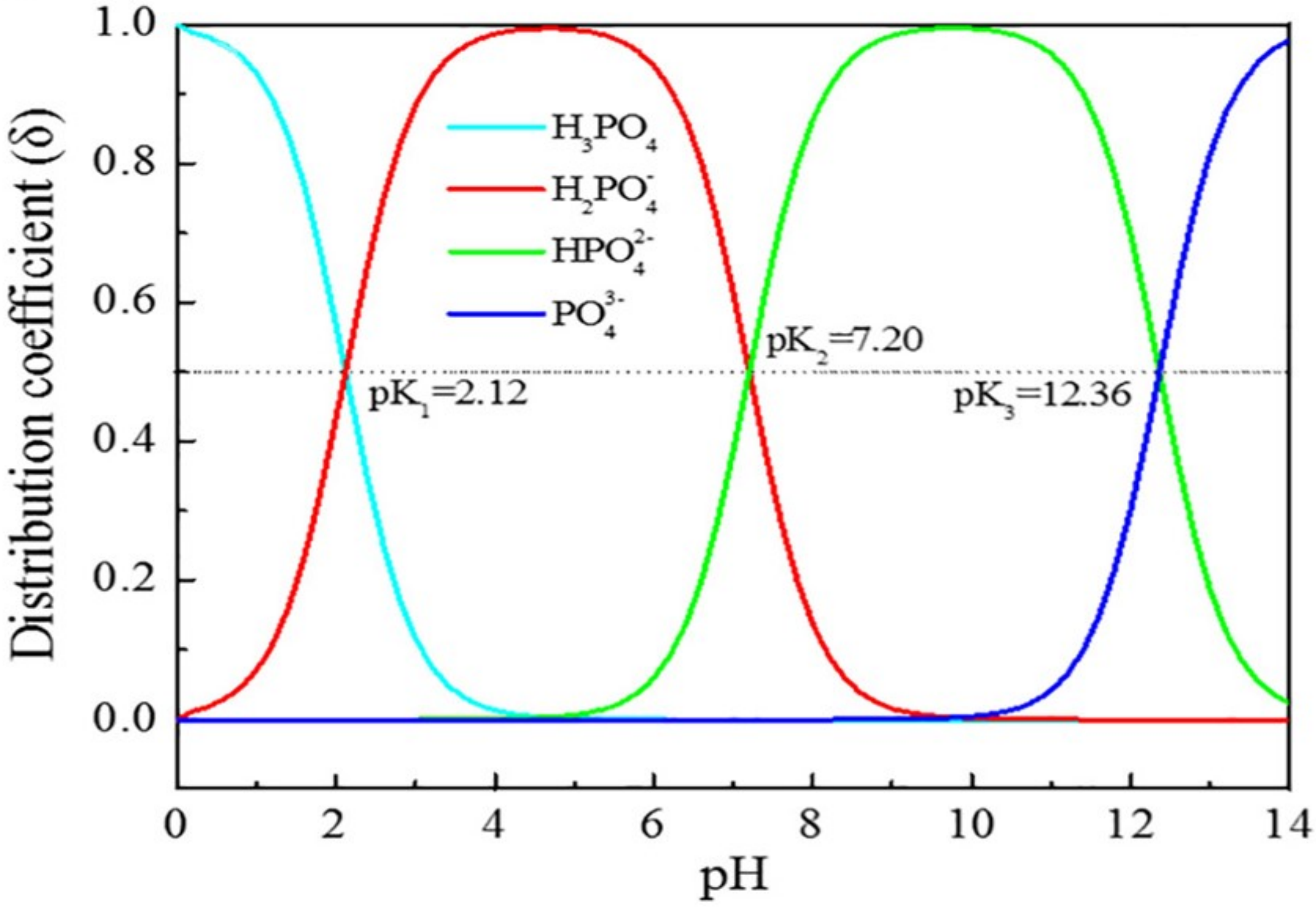

Figure S7. Influence of pH on the distribution of phosphate species, adapted from Zhang et al., 2019

References

Belbali, A., Benghalem, A., Gouttal, K., Taleb, S., & Ziri, F. Z. (2022). Humic acid removal from water using Moringa protein functionalized biochar husk. *Desalination and Water Treatment*, *279*, 29–35. https://doi.org/10.5004/dwt.2022.29064

Bezza, F. A., Brink, H. G., & Chirwa, E. M. N. (2024). Selective and efficient removal of phosphate from aqueous solution using activated carbon-supported Mg–Fe layered double oxide nanocomposites. *Canadian Journal of Chemical Engineering*. https://doi.org/10.1002/cjce.25440

Bolbol, H., Fekri, M., & Hejazi-Mehrizi, M. (2019). Layered double hydroxide–loaded biochar as a sorbent for the removal of aquatic phosphorus: behavior and mechanism insights. *Arabian Journal of Geosciences*, *12*(16). https://doi.org/10.1007/s12517-019-4694-4

Davoudli, H. K., Saadat, S., Rahsepar, M., & Azadi, F. (2021). Investigating the role of the morphology of the Zn-Al LDH on the adsorption of humic acid from aqueous solutions. *Water Science and Technology*, *84*(7), 1663–1677. https://doi.org/10.2166/wst.2021.373

George, R., & Sugunan, S. (2014). Kinetics of adsorption of lipase onto different mesoporous materials: Evaluation of Avrami model and leaching studies. *Journal of Molecular Catalysis B: Enzymatic*, *105*, 26–32. https://doi.org/10.1016/j.molcatb.2014.03.008

Huang, W., Chen, J., He, F., Tang, J., Li, D., Zhu, Y., & Zhang, Y. (2015). Effective phosphate adsorption by Zr/Al-pillared montmorillonite: Insight into equilibrium, kinetics and thermodynamics. *Applied Clay Science*, *104*, 252–260. https://doi.org/10.1016/j.clay.2014.12.002

Jing, H. P., Li, Y., Wang, X., Zhao, J., & Xia, S. (2019). Simultaneous recovery of phosphate, ammonium and humic acid from wastewater using a biochar supported Mg(OH)2/bentonite composite. *Environmental Science: Water Research and Technology*, *5*(5), 931–943. https://doi.org/10.1039/c8ew00952j

Lima, É. C., Adebayo, M. A., & Machado, F. M. (2015). Kinetic and equilibrium models of adsorption. In *Carbon Nanostructures* (Vol. 0, Issue 9783319188744, pp. 33–69). Springer International Publishing. https://doi.org/10.1007/978-3-319-18875-1_3

Mahfoudhi, S., Mansour, A. ben, Hammouda, S. ben, Hafiane, A., & Trabelsi, I. (2022). Optimization of humic acid adsorption using central composite design (CCD) and principal component analysis (PCA): kinetics, isotherm, and thermodynamics studies. *Arabian Journal of Geosciences*, *15*(10). https://doi.org/10.1007/s12517-022-10202-6

Noorimotlagh, Z., Ravanbakhsh, M., Valizadeh, M. R., Bayati, B., Kyzas, G. Z., Ahmadi, M., Rahbar, N., & Jaafarzadeh, N. (2020). Optimization and genetic programming modeling of humic acid adsorption onto prepared activated carbon and modified by multi-wall carbon nanotubes. *Polyhedron*, *179*. https://doi.org/10.1016/j.poly.2020.114354

Novais, S. V., Zenero, M. D. O., Tronto, J., Conz, R. F., & Cerri, C. E. P. (2018). Poultry manure and sugarcane straw biochars modified with MgCl2 for phosphorus adsorption. *Journal of Environmental Management*, *214*, 36–44. https://doi.org/10.1016/j.jenvman.2018.02.088

Pap, S., Kirk, C., Bremner, B., Turk Sekulic, M., Shearer, L., Gibb, S. W., & Taggart, M. A. (2020). Low-cost chitosan-calcite adsorbent development for potential phosphate removal and recovery from wastewater effluent. *Water Research*, *173*. https://doi.org/10.1016/j.watres.2020.115573

Rashtbari, Y., Arfaeinia, H., Ahmadi, S., Bahrami Asl, F., Afshin, S., Poureshgh, Y., & Fazlzadeh, M. (2022). Potential of using green adsorbent of humic acid removal from aqueous solutions: equilibrium, kinetics, thermodynamic and regeneration studies. *International Journal of Environmental Analytical Chemistry*, *102*(17), 5373–5390. https://doi.org/10.1080/03067319.2020.1796993

Salem, D. ben, Ouakouak, A., Touahra, F., Hamdi, N., Eltaweil, A. S., Syed, A., Boopathy, R., & Tran, H. N. (2023). Easy separable, floatable, and recyclable magnetic-biochar/alginate bead as super-adsorbent for adsorbing copper ions in water media. *Bioresource Technology*, *383*. https://doi.org/10.1016/j.biortech.2023.129225

Tran, H. N., You, S. J., Hosseini-Bandegharaei, A., & Chao, H. P. (2017). Mistakes and inconsistencies regarding adsorption of contaminants from aqueous solutions: A critical review. In *Water Research* (Vol. 120, pp. 88–116). Elsevier Ltd. https://doi.org/10.1016/j.watres.2017.04.014

Tran, H. N. (2022). Improper Estimation of Thermodynamic Parameters in Adsorption Studies with Distribution Coefficient K D(q e/ C e) or Freundlich Constant (K F): Considerations from the Derivation of Dimensionless Thermodynamic Equilibrium Constant and Suggestions. *Adsorption Science and Technology*, *2022*. https://doi.org/10.1155/2022/5553212

Van Truong, T., Kim, Y. J., & Kim, D. J. (2023). Study of biochar impregnated with Al recovered from water sludge for phosphate adsorption/desorption. *Journal of Cleaner Production*, *383*. https://doi.org/10.1016/j.jclepro.2022.135507

Yan, X., Du, W., Ma, C., Cheng, S., & Li, X. (2020). Humic acid adsorption behavior and mechanism comparison between biochars and activated carbon. *Desalination and Water Treatment*, *173*, 213–222. https://doi.org/10.5004/dwt.2020.24825

Yue, D., Wang, J., Cui, D., Zhang, L., & Dong, X. (2021). Insights into adsorption of humic substances on graphitic carbon nitride. *Environmental Science and Technology*, *55*(12), 7910–7919. https://doi.org/10.1021/acs.est.0c07681

Zhang, A., Chen, W., Gu, Z., Li, Q., & Shi, G. (2018). Mechanism of adsorption of humic acid by modified aged refuse. *RSC Advances*, *8*(59), 33642–33651. https://doi.org/10.1039/C8RA05933K

Zhang, Z., Yan, L., Yu, H., Yan, T., & Li, X. (2019). Adsorption of phosphate from aqueous solution by vegetable biochar/layered double oxides: Fast removal and mechanistic studies. *Bioresource Technology*, *284*, 65–71. https://doi.org/10.1016/j.biortech.2019.03.113
